# Altered binding affinity of SIX1-Q177R correlates with enhanced *WNT5A* and WNT pathway effector expression in Wilms tumor

**DOI:** 10.1242/dmm.050208

**Published:** 2023-11-17

**Authors:** Matthew J. Stevenson, Sabrina K. Phanor, Urvi Patel, Stephen S. Gisselbrecht, Martha L. Bulyk, Lori L. O'Brien

**Affiliations:** ^1^ Department of Cell Biology and Physiology, University of North Carolina at Chapel Hill, Chapel Hill, NC 27599, USA; ^2^ Division of Genetics, Department of Medicine, Brigham and Women's Hospital and Harvard Medical School, Boston, MA 02115, USA; ^3^ Department of Pathology, Brigham and Women's Hospital and Harvard Medical School, Boston, MA 02115, USA

**Keywords:** Wilms tumor, Kidney development, SIX1, WNT signaling, Gene regulation

## Abstract

Wilms tumors present as an amalgam of varying proportions of tissues located within the developing kidney, one being the nephrogenic blastema comprising multipotent nephron progenitor cells (NPCs). The recurring missense mutation Q177R in NPC transcription factors SIX1 and SIX2 is most correlated with tumors of blastemal histology and is significantly associated with relapse. Yet, the transcriptional regulatory consequences of SIX1/2-Q177R that might promote tumor progression and recurrence have not been investigated extensively. Utilizing multiple Wilms tumor transcriptomic datasets, we identified upregulation of the gene encoding non-canonical WNT ligand WNT5A in addition to other WNT pathway effectors in SIX1/2-Q177R mutant tumors. SIX1 ChIP-seq datasets from Wilms tumors revealed shared binding sites for SIX1/SIX1-Q177R within a promoter of *WNT5A* and at putative distal cis-regulatory elements (CREs). We demonstrate colocalization of SIX1 and WNT5A in Wilms tumor tissue and utilize *in vitro* assays that support SIX1 and SIX1-Q177R activation of expression from the *WNT5A* CREs, as well as enhanced binding affinity within the *WNT5A* promoter that may promote the differential expression of *WNT5A* and other WNT pathway effectors associated with SIX1-Q177R tumors.

## Teaser

**Summary:** By using novel methods to compare Wilms tumor genomic datasets and *in vitro* assays, we identified aberrant gene expression programs linked to enhanced DNA-binding affinity that result from the relapse-associated Q177R mutation in SIX1 and SIX2.

## INTRODUCTION

Wilms tumor, the most common childhood kidney cancer is typically diagnosed between 2–5 years of age and accounts for ∼5% of all cancers in patients under the age of 14 (Steliarova-Foucher et al., 2017; Breslow et al., 2006; Hol et al., 2019). The histological composition of Wilms tumors resembles that of the normal developing human fetal kidney (hFK), comprising varying degrees of blastemal, epithelial and stromal tissues. Each of these tissues is thought to represent distinct compartments within the developing kidney, i.e. (1) nephron progenitor cells (NPCs), the multipotent progenitor population that gives rise to all epithelial cell types of the nephron; (2) differentiating/differentiated epithelial tubules and; (3) interstitial cells (ICs), respectively (reviewed by Rivera and Haber, 2005). Beyond the morphological similarities, numerous microarray and transcriptomic analyses have revealed gene expression signatures in Wilms tumors that resemble those of normal pre- and post-induction NPCs with varying degrees of differentiation. These include the conserved expression of genes encoding for core NPC transcription factors known to be required for the specification and/or maintenance of NPCs, such as *SIX2*, *PAX2* and *SALL1* (Kobayashi et al., 2008; Park et al., 2012; Self et al., 2006; Naiman et al., 2017; Kanda et al., 2014; Huang et al., 2016; Basta et al., 2014), altogether implying that stalled nephrogenesis underlies formation of these tumors (Li et al., 2002; Fukuzawa et al., 2017; Wegert et al., 2015; Walz et al., 2015; Gadd et al., 2012, 2017; Young et al., 2018; Trink et al., 2018). Moreover, a study using mice in which Wilms tumor-associated mutations – including loss of *Wt1* with concurrent stabilization of β-catenin, or loss of *Wt1* with concurrent biallelic expression of *Igf2* – had been introduced specifically in stromal or NPC lineages demonstrated that these mutation combinations only result in tumor formation within the NPC lineage, further supporting the nephrogenic origin of these tumors (Huang et al., 2016). Despite the apparent morphological and molecular similarities to hFK and the characteristically low mutational burden of Wilms tumors and pediatric malignancies in general compared to that of adult cancers, the breadth of molecular mechanisms underlying propagation and recurrence of these tumors remains unclear (Wegert et al., 2015; Gröbner et al., 2018; Kandoth et al., 2013).

Treatment of Wilms tumor is generally considered a success with a 5-year relative survival over 90% [Surveillance, Epidemiology, and End Results (SEER) cancer statistics 1975–2017; https://seer.cancer.gov/archive/csr/1975_2017/], yet blastemal predominant tumors continue to present a challenge to therapeutic intervention. Between 2001 and 2012, 20% of patients with blastemal predominant tumors treated according to International Society of Paediatric Oncology protocols, consisting of neoadjuvant chemotherapy followed by kidney resection, relapsed within 5 years of diagnosis and 95% of relapses were distant metastases (Van den Heuvel-Eibrink et al., 2015). In patients treated according to the Children's Oncology Group (COG) guidelines, in which kidney resection is performed prior to chemotherapy/radiotherapy, 88% of relapses of stage III tumors occurred within the first 2 years after diagnosis, including five of seven blastemal tumors, as analyzed in a study by Fernandez et al. (2018). Five-year overall survival after relapse of favorable histology Wilms tumor (FHWT) – classified by the COG as tumors that lack evidence of diffuse anaplasia – is estimated to be 60–70% (Mullen et al., 2018), highlighting the need for improved therapies to limit the risk of relapse for Wilms tumors.

Mutations in the NPC-associated transcription factors *SIX1* and *SIX2* (∼7%) are most associated with blastemal predominant tumors (Gadd et al., 2017; Walz et al., 2015; Wegert et al., 2015). Of the variants detected in *SIX1* across several studies, the overwhelming majority were a glutamine to arginine substitution, p.Q177R (hereafter referred to as Q177R). This mutation is significantly associated with relapse, having recently been identified in ∼13% of relapsed tumors (Gadd et al., 2022). Wegert et al. (2015) provided the first mechanistic investigation of the SIX1-Q177R mutant protein by using ChIP-seq, demonstrating a shift in the DNA-binding motif for SIX1-Q177R when compared to that of wild-type SIX1 in primary Wilms tumor tissues. Increased binding of SIX1-Q177R was observed near the *TGFA* gene with corresponding elevated expression of *TGFA* in SIX1-Q177R mutant tumors compared to tumors comprising wild-type SIX1 (Wegert et al., 2015). More broadly, however, the consequences of this altered DNA binding on downstream transcriptional regulatory networks, and its potential role in facilitating tumor progression, chemotherapeutic resistance and relapse, remain unexplored.

During kidney development, a balance of NPC self-renewal versus differentiation ensures that a full complement of nephrons is formed prior to NPC exhaustion near birth. This is accomplished through a complex interplay of developmental signaling pathways, including fibroblast growth factor, bone morphogenetic protein and WNT pathways, as well as transcriptional mechanisms (reviewed by O'Brien, 2019). With roles in self-renewal as well as differentiation, the WNT pathway contributes to this balance through both canonical/β-catenin dependent and non-canonical/β-catenin independent mechanisms. It has been shown that low levels of canonical WNT/β-catenin-mediated signaling support NPC self-renewal (Karner et al., 2011; Ramalingam et al., 2018). In contrast, high levels of canonical WNT/β-catenin-mediated signaling induce differentiation of NPCs (Carroll et al., 2005; Park et al., 2007; Ramalingam et al., 2018). Non-canonical WNT signals also support maintenance of the NPC niche to ensure sufficient nephron endowment (O'Brien et al., 2018b). Together, these findings highlight the balance of appropriate WNT signals required for proper nephrogenesis and suggest how dysregulation could lead to, or support, inappropriate development and disease.

In mice, the homeobox transcription factor Six2 is required to maintain NPC self-renewal (Self et al., 2006; Kobayashi et al., 2008). In addition, the closely related transcription factor Six1 is required for kidney development in mouse, as knockout results in kidney agenesis. Six1 also acts upstream of canonical NPC transcription factors – including Six2 and Pax2 – placing it near the top of the NPC gene regulatory network hierarchy. While Six1 acts early in the metanephric mesenchyme (MM), the precursor population to NPCs, to support the expression of these factors it is rapidly downregulated in the NPCs (Xu et al., 2003; Li et al., 2003). Comparative investigations of embryonic mouse and hFK development has demonstrated prolonged temporal expression of *SIX1*/SIX1 in human NPCs through later stages of development, in addition to novel regulatory interactions between SIX1 and SIX2 not observed in mouse (O'Brien et al., 2016). These observations suggest that SIX1 may also support NPC self-renewal during human nephrogenesis, representing an expanded regulatory role for SIX1 that may underlie its frequent mutation and association with relapsed Wilms tumors.

The goal of this study was to expand upon previous reports and more clearly define the regulatory role of SIX1-Q177R in Wilms tumor by utilizing genomic data available from large-scale studies of these tumors in tandem with SIX1 ChIP-seq data from normal hFK. In doing so and when compared to other blastemal tumors, we identified upregulated expression of the gene encoding the non-canonical WNT ligand WNT5A in both chemotherapy-naïve and chemotherapy-treated blastemal Wilms tumors harboring the SIX1-Q177R mutation. Furthermore, we identified upregulation of several WNT pathway effectors in SIX1-Q177R tumors compared to other chemotherapy-naïve blastemal tumors, including positive regulators of non-canonical WNT signaling and negative regulators of canonical WNT/β-catenin-mediated signaling, implicating disruption of this signaling pathway in the maintenance and therapeutic evasion of SIX1-Q177R Wilms tumors. We illustrate the colocalization of SIX1 and WNT5A proteins within individual cells in Wilms tumor tissue by immunofluorescence, demonstrate SIX1 and SIX1-Q177R enhancement of transcription via putative *WNT5A* cis-regulatory elements (CREs) *in vitro*, and provide a mechanistic link to *WNT5A* upregulation that is attributable to the enhanced DNA-binding affinity of SIX1-Q177R within the *WNT5A* promoter. These findings shed new light on the disrupted gene regulatory networks associated with SIX1/2-Q177R, which might disturb the balance of WNT signaling in NPCs, thereby perpetuating escape from differentiation to promote tumor propagation, recurrence and, potentially, facilitate tumorigenesis.

## RESULTS

### Blastemal histology and SIX1/2-Q177R are not associated with upregulation of core NPC transcription factor genes in chemotherapy-naïve Wilms tumors

To begin addressing the regulatory consequences associated with SIX1-Q177R, we performed differential gene expression (DGE) analyses utilizing mRNA microarray data generated from 42 chemotherapy-treated tumors (Wegert et al., 2015) as well as RNA-seq data from 86 chemotherapy-naïve high-risk FHWTs of varying histology classifications that, subsequently, had relapsed, generated as part of the Therapeutically Applicable Research to Generate Effective Treatments (TARGET) program (Walz et al., 2015). The same Q177R mutation has been identified in SIX2 in Wilms tumors but occurs almost half as frequently as SIX1-Q177R (Wegert et al., 2015; Walz et al., 2015; Gadd et al., 2017). SIX2 shares ∼100% amino acid residue conservation within the DNA-binding homeodomain, binds most of the same genomic sites and targets ∼90% of the genes targeted by SIX1 in hFK (O'Brien et al., 2016). Significantly, separation of SIX1-Q177R and SIX2-Q177R tumors did not alter the primary findings of our DGE analysis (Table S1). Accounting for these similarities between SIX1 and SIX2, and to increase the statistical power of our analyses, we grouped SIX1-Q177R and SIX2-Q177R tumors (hereafter referred to as SIX1/2-Q177R) in each dataset.

Almost 30% of SIX1/2-Q177R tumors also harbor inactivating mutations in the microRNA (miRNA)-processing genes *DROSHA* or *DGCR8* (Wegert et al., 2015; Walz et al., 2015; Gadd et al., 2017). Co-occurrence of SIX1/2-Q177R and *DROSHA*/*DGCR8* mutations significantly increased the rates of relapse as well as death in a synergistic manner, compared to tumors with other mutations and tumors with SIX1/2-Q177R or *DROSHA*/*DGCR8* mutations alone (Walz et al., 2015). Yet, targeted mutation or deletion of *Drosha* within NPCs does not result in tumor formation in mice (Kruber et al., 2018). Furthermore, Wegert et al. observed no strong effects on gene expression in *DROSHA*/*DGCR8* mutant tumors, only a significant reduction in miRNA levels (Wegert et al., 2015). Accordingly, miRNA processing gene mutations were not used for further sample stratification.

Specific histology classification of other tumors used in this analysis was based on prior classification if provided [(see Wegert et al., 2015; Walz et al., 2015; and the TARGET data matrix (https://portal.gdc.cancer.gov/projects/TARGET-WT)], otherwise tumors were included in the groups containing tumors of mixed/epithelial/stromal/regressive histology. Of note, although SIX1/2-Q177R is most associated with blastemal histology (Wegert et al., 2015; Walz et al., 2015), three of the SIX1/2-Q177R tumors in the RNA-seq analysis were classified as mixed histology (Walz et al., 2015; Gadd et al., 2017). However, removal of those tumors from the SIX1/2-Q177R group did not alter the primary findings of the DGE analysis (Table S1). Owing to the overwhelming evidence supporting the nephrogenic origin of Wilms tumors (reviewed by Li et al., 2021 and Hohenstein et al., 2015; Huang et al., 2016; Coorens et al., 2019), we reasoned an approach for the RNA-seq analysis focusing on transcripts expressed in all tumor samples could elucidate important deviations along the nephrogenic trajectory between tumors, while also highlighting crucial intrinsic characteristics shared between these tumors. Therefore, weakly expressed genes were stringently filtered to exclude potential false positives that had been due to heterogeneity in tumor microenvironments or sample-to-sample processing variability, resulting in the removal of 80% of genes from this analysis.

As described by Wegert et al., blastemal type tumors displayed upregulated cell proliferation and kidney progenitor gene signatures compared to tumors of other histology (Wegert et al., 2015). These upregulated genes included core NPC transcription factors that, however, were not further upregulated in SIX1/2-Q177R tumors when compared to other blastemal tumors in this dataset, suggesting this mutation does not exert its effect through enhancement of the NPC transcriptional regulatory program (Table S1). Surprisingly, in the chemotherapy-naïve RNA-seq datasets we did not identify similar upregulated signatures between blastemal and mixed/epithelial/stromal tumors. However, several of these core NPC transcription factors did not meet the stringent expression threshold we set for our analysis. As such, relaxation of this threshold resulted in the inclusion of these genes, yet revealed no evidence for differential expression of *SIX1*, *SIX2*, *PAX2*, *SALL1*, *WT1*, *HOXA/C/D11* and *OSR1* (Table S1) (Donovan et al., 1999; Mugford et al., 2008a,b; Xu et al., 2014). Similarly, there was no evidence of significant differential expression of any of these genes in SIX1/2-Q177R tumors, demonstrating that enhanced expression of core NPC transcription factors is neither intrinsic to blastemal Wilms tumors nor a consequence of SIX1/2-Q177R.

### Elevated expression of *WNT5A* in both chemotherapy-naïve and chemotherapy-treated blastemal tumors is associated with SIX1/2-Q177R

**Fig. 1.**
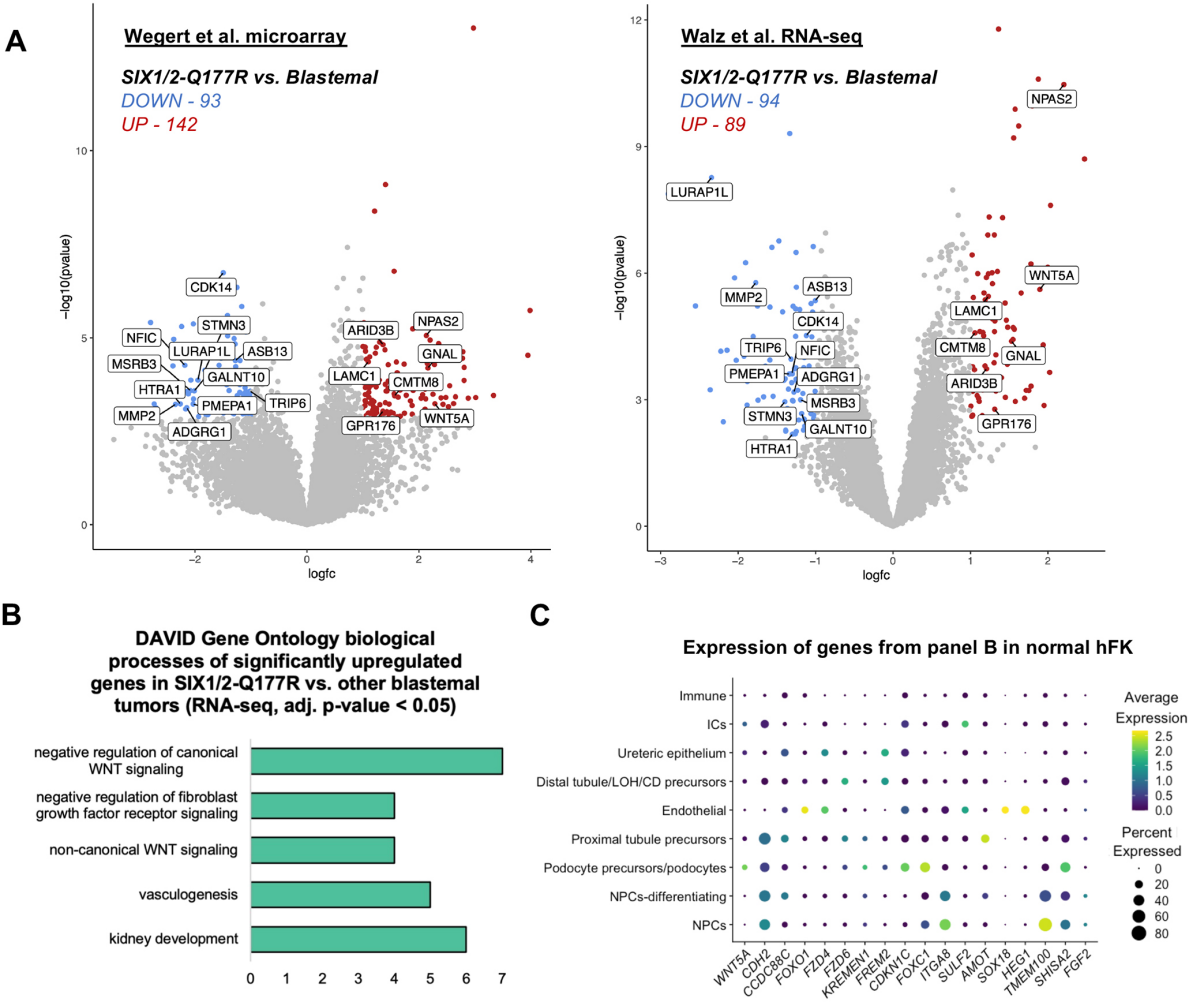
***WNT5A* and other WNT pathway effectors are upregulated in SIX1/2-Q177R Wilms tumors compared to other blastemal tumors.** (A) Volcano plots depicting results from DGE analyses of Wilms tumor microarray and RNA-seq datasets comparing SIX1/2-Q177R Wilms tumors to other blastemal Wilms tumors. Genes significantly upregulated are shown in red; genes significantly downregulated are shown in blue. Labeled genes indicate those found to be significantly differentially expressed in both datasets. Significant differential expression was defined as log_2_-fold change ≥|1.0| with adjusted *P*-value <0.05 (false discovery rate). Microarray: SIX1/2-Q177R *n*=4; Blastemal *n*=23. RNA-seq: SIX1/2-Q177R *n*=8; Blastemal *n*=22. (B) Bar chart showing the number of genes significantly upregulated in SIX1/2-Q177R tumors compared to that in other blastemal tumors within the RNA-seq dataset, annotated in each of the shown Gene Ontology Biological Process terms identified by using the Database for Annotation, Visualization and Integrated Discovery (DAVID). (C) Dot plot generated from integrated hFK single-cell RNA-seq datasets (see Materials and Methods) displaying scaled average expression of all cells within the indicated cell clusters and the percent of cells in each cluster expressing the indicated genes that contributed to the GO terms shown in panel B. LOH, loop of Henle, CD, collecting duct.

Seven genes were identified as significantly upregulated in SIX1/2-Q177R tumors compared to other blastemal tumors in both datasets (log_2_-fold change≥|1.0|, adjusted *P* value <0.05): *WNT5A*, *GNAL*, *NPAS2*, *CMTM8*, *GPR176*, *LAMC1* and *ARID3B* (Fig. 1A). Of particular interest – as it relates to potential chemotherapeutic resistance and increased rates of relapse of SIX1/2-Q177R tumors – is *WNT5A*, a non-canonical WNT signaling pathway ligand. WNT5A has been associated with increased resistance to chemotherapeutics in ovarian and breast cancer cells (Peng et al., 2011; Hung et al., 2014), and approaches of directly targeting WNT5A-mediated signaling for cancer therapy have been proposed for prostate cancer (Gao et al., 2022), small cell lung cancer (Kim et al., 2022) and melanoma (Jenei et al., 2009).

Wnt5a has been shown to play a role in the earliest stages of mouse kidney development, mediating generation of the MM through regulation of proper extension of the posterior intermediate mesoderm, the cell population that gives rise to the MM, as well as regulation of cell proliferation (Huang et al., 2014; Nishita et al., 2014; Yun et al., 2014). *In situ* hybridization revealed expression of *Wnt5a* in the intermediate mesoderm at E9.5 and in the MM, albeit weakly, up to E11.5 (Nishita et al., 2014; Yun et al., 2014). This developmental window coincides with the expression of Six1 in the intermediate mesoderm/MM as discussed earlier, raising the possibility of transcriptional regulation of *Wnt5a* by Six1 during normal kidney development. In the context of Wilms tumor, an investigation of chromatin profiles included *WNT5A* in a set of genes alongside core NPC genes *SIX2*, *GDNF*, *EYA1* and *OSR1* that were characterized by broad H3K4me3 domains (>3.5 kb) within 2.5 kb of the gene promoter in Wilms tumors, illustrating that the *WNT5A* promoter/gene is typically active in these tumors (Aiden et al., 2010). Furthermore, Wnt5a has been shown to inhibit canonical WNT/β-catenin signaling (Topol et al., 2003; Mikels and Nusse, 2006), which could aid in promoting the Wilms tumorigenic program by interfering with normal differentiation of NPCs, thus warranting further investigation.

### WNT pathway effectors are upregulated in SIX1/2-Q177R Wilms tumors compared to other blastemal tumors

Gene Ontology (GO) enrichment analysis by using the Database for Annotation, Visualization and Integrated Discovery (DAVID) (Sherman et al., 2022; Huang et al., 2009) revealed significant enrichment of biological processes such as ‘non-canonical WNT signaling’ and ‘negative regulation of canonical WNT signaling’ associated with the SIX1/2-Q177R upregulated genes compared to other blastemal tumors identified in the RNA-seq dataset including: *WNT5A*, *FZD4*, *FZD6*, *CCDC88C*, *CDH2*, *FOXO1* and *KREMEN1* (Fig. 1B; Table S1). By examining the upregulated genes more closely through literature searches, additional proteins with functions either promoting non-canonical WNT signaling and/or antagonizing canonical WNT/β-catenin signaling became apparent. Positive regulators of non-canonical WNT signaling include IQGAP2 through interaction with Cdc42/Rac1 (Logue et al., 2011; Ozdemir et al., 2018; Fukata et al., 2002), ARHGEF3 through activation of RhoA (D'Amato et al., 2015; You et al., 2021), and FOXC1 through direct regulation of *WNT5A* expression (Han et al., 2017). Negative regulators of canonical WNT/β-catenin signaling include KDM2B through independent demethylation of β-catenin and transcriptional repression of β-catenin target genes (Lu et al., 2015; Lađinović et al., 2020), as well as SLIT3 and IQGAP2, as the expression of both has been associated with decreased β-catenin target gene activation or decreased nuclear β-catenin, respectively (Kim et al., 2018; Ng et al., 2018; Deng et al., 2015). These observations highlight a potential imbalance in WNT pathway signaling associated with SIX1/2-Q177R.

### SIX1, WNT5A and canonical podocyte lineage proteins colocalize in Wilms tumor

GO analysis also identified the biological process ‘kidney development’ as being enriched among the upregulated genes (Fig. 1B). To identify the expression domains of these and the other upregulated genes contributing to the GO terms within the normal developing human kidney, we integrated available hFK single-cell RNA-seq datasets for convenient visualization (Fig. S1A) (Lindström et al., 2018a, 2021; Tran et al., 2019). Expression of *WNT5A* was highest in podocyte precursors/podocytes; no detectable expression was identified in NPCs (Fig. 1C). This is in agreement with *in situ* hybridization evidence demonstrating podocyte expression of *WNT5A* in hFK (Fig. S1B) (McMahon, 2019; https://www.gudmap.org/id/16-QME2). Podocytes are highly specialized epithelial derivatives of NPCs, forming extensive mesh-like networks with one another in which cellular protrusions termed ‘foot processes’ interdigitate and surround the glomerular capillaries to filter the incoming blood (reviewed by Garg, 2018). Interestingly, *KREMEN1*, *SHISA2*, *CDKN1C* and *FOXC1* also show enriched expression in podocyte precursors/podocytes (Fig. 1C). In addition, Sulf2 has been shown to play a role in mature podocytes, contributing to the structural integrity of the glomerular filtration barrier (Schumacher et al., 2011; Takashima et al., 2016). Also included in this upregulated gene set are *ARHGEF3* and *IQGAP2*, both of which display podocyte-enriched expression in the developing hFK (Fig. S1C). Apart from podocyte precursors/podocytes, genes enriched in precursors of other nephron segments – including proximal and distal tubules (*CDH2, CCDC88C, FZD6, FREM2, AMOT*) as well as NPCs (*CDH2, CCDC88C, FOXC1, ITGA8, TMEM100, FGF2*) – were also upregulated in SIX1/2-Q177R tumors (Fig. 1C; Fig. S1C). Altogether, these data further underscored the nephrogenic origin of these tumors.

**Fig. 2.**
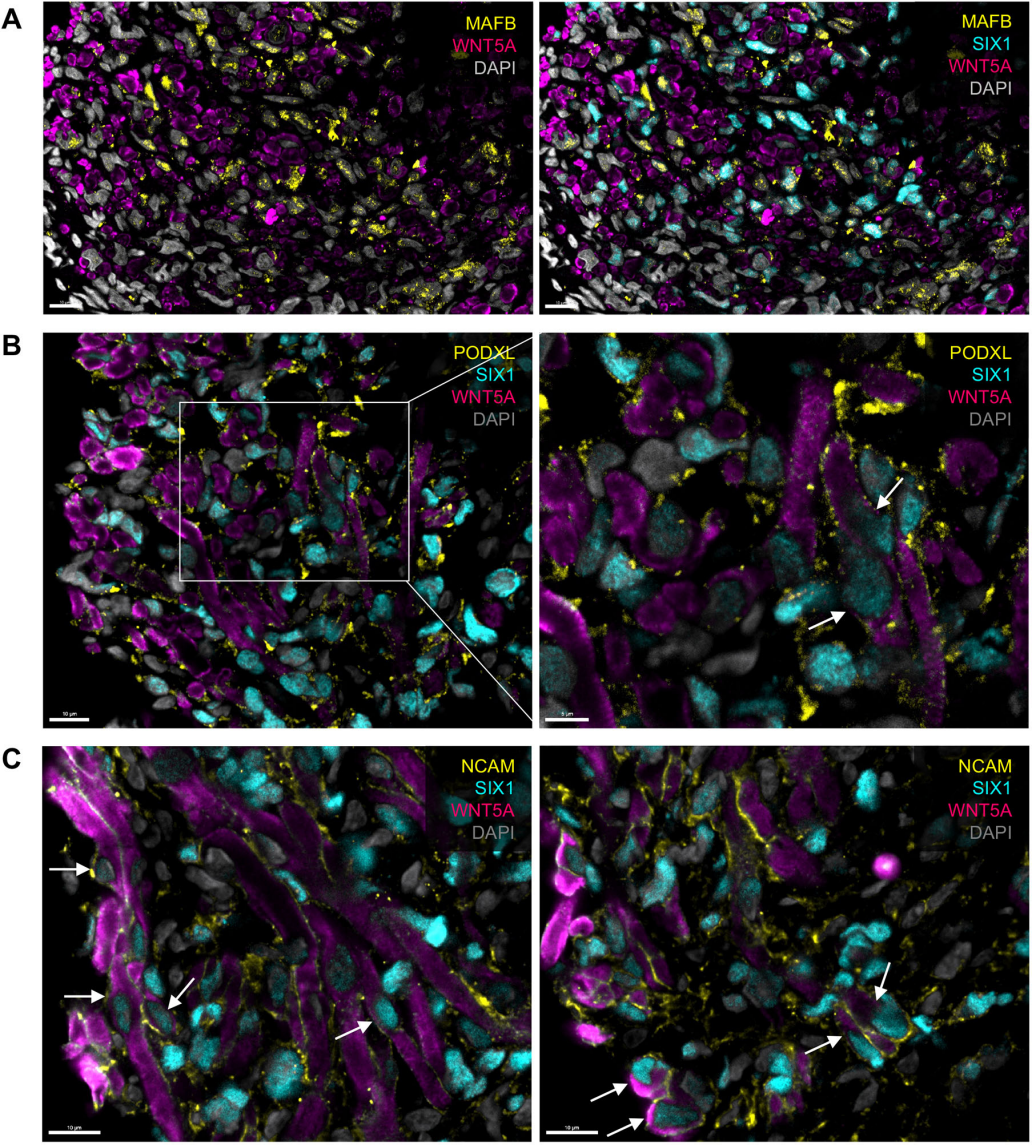
**SIX1 and WNT5A colocalize in Wilms tumor cells along with podocyte lineage proteins.** Immunofluorescence images of Wilms tumor tissue. (A) Staining for MAFB and WNT5A, and their overlap with SIX1 (right). (B) Staining for PODXL, SIX1 and WNT5A in Wilms tumor tissue. The boxed area is shown magnified on the right, with arrows indicating examples of colocalization of SIX1 and WNT5A proteins within individual cells. (C) Staining for NCAM, SIX1 and WNT5A, with arrows indicating SIX1 and WNT5A colocalization within individual cells (shown in two separate images). All scale bars: 10 µm (except panel B right image, where scale bar: 5 µm). Nuclei were stained with DAPI.

As Wilms tumors are widely characterized by their resemblance to the normal developing kidney, the prevalence of podocyte precursor/podocyte-enriched genes is notable considering the sparse characterization of podocyte lineage marker expression in previous studies of Wilms tumors. Given the availability of robust antibodies against established podocyte markers MAFB (transcription factor) and PODXL (cell surface protein) (Tran et al., 2019), both of which were found to be expressed in all tumors within the RNA-seq dataset (Table S1), we assessed the presence and localization of these markers in addition to SIX1 and WNT5A by immunofluorescence in Wilms tumor tissue of mixed histology and unknown mutational status. WNT5A cytosolic/membranous staining was observed in areas of tissue containing MAFB- and SIX1-positive nuclei, as well as PODXL-positive cells (Fig. 2A,B). Expression of *MAFB* and *PODXL* has been reported in Wilms tumor cell lines and expression of *PODXL* has been observed in prior microarray analyses of Wilms tumors of varying histology (Royer-Pokora et al., 2023; Trink et al., 2018). Nevertheless, to our knowledge, our findings provide the first evidence of the podocyte lineage markers MAFB and PODXL at protein level in Wilms tumor. Significantly, closer examination  − using NCAM and PODXL as markers of cell membrane − confirmed the presence of SIX1 and WNT5A double-positive cells (Fig. 2B,C). NCAM has been proposed as a marker of a subset of cancer stem cells or cancer-initiating cells within Wilms tumors, particularly in association with propagation of the blastemal compartment (Pode-Shakked et al., 2008, 2012; Shukrun et al., 2014). However, immunohistochemical studies of Wilms tumors have demonstrated localization of both SIX1 and NCAM in all histological compartments (Kapur and Rakheja, 2011; Sehic et al., 2014). Therefore, classification of the SIX1/WNT5A double-positive cells would require the characterization of a larger panel of markers by immunostaining or single-cell transcriptomic analysis. Nonetheless, together with our finding of specific upregulation of *WNT5A* in tumors harboring the SIX1/2-Q177R mutation, this observation indicates that *WNT5A* may be a direct regulatory target of SIX1/SIX1-Q177R in Wilms tumor.

### The Q177R mutation alone alters the DNA-binding preference of SIX1

To address the possibility of *WNT5A* regulation by SIX1, we turned to available SIX1 ChIP-seq data generated from wild-type SIX1 and SIX1-Q177R mutant Wilms tumors (Wegert et al., 2015). In that study, the authors identify a differential DNA-binding motif for SIX1-Q177R in addition to enhanced expression of the putative target gene *TGFA*. However, additional regulatory targets and GO enrichment analyses has not been reported and it is unknown how these would compare to those of SIX1 in hFK (Wegert et al., 2015; O'Brien et al., 2016). Therefore, we aimed to conduct expanded and novel comparisons to interrogate SIX1 regulatory programs in Wilms tumor, particularly those associated with the SIX1-Q177R mutation. However, as the SIX1-Q177R ChIP-seq data had been obtained from a single chemotherapy-treated Wilms tumor (Wegert et al., 2015), we first sought to investigate the DNA-binding preference of the mutant protein in the absence of potentially confounding variables, including tissue quality, chemotherapy-induced artefacts and interacting protein co-factors that can shift the DNA-binding preference of transcription factors (Siggers et al., 2011; Slattery et al., 2011).

**Fig. 3.**
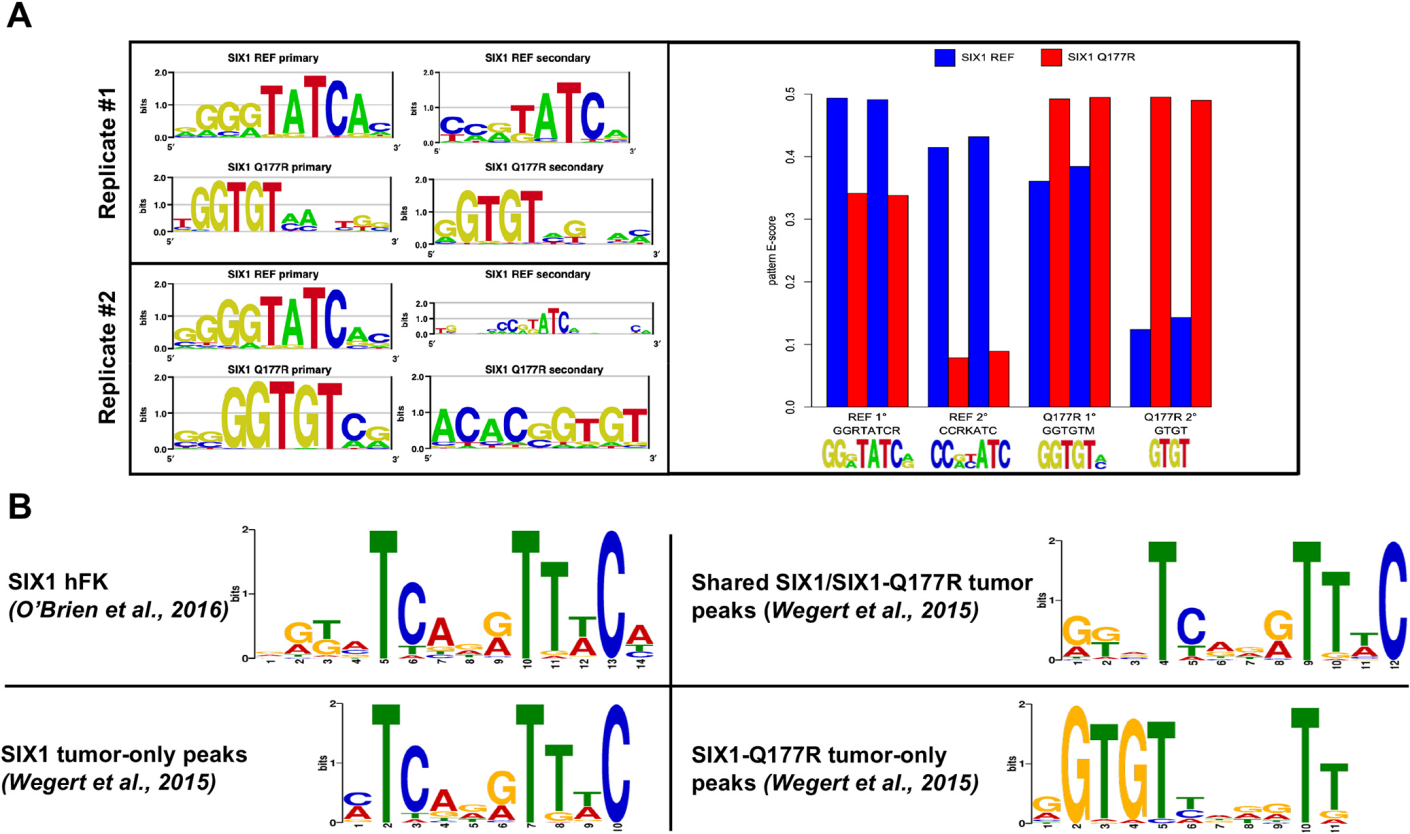
**The Q177R variant alone modifies the primary DNA sequence preference of SIX1.** (A) Left: Representative sequence logos of the primary binding preferences of the SIX1 reference allele (top in each replicate) and Q177R variant (bottom in each replicate), and of the additional (secondary) binding specificity. Right: Pattern E-scores (see Materials and Methods) for binding of the SIX1 reference allele (blue) and Q177R variant (red) to each indicated pattern. (B) SIX1- and SIX1-Q177R DNA-binding motifs as discovered by using the STREME tool (see Materials and Methods) from ChIP-seq datasets of Wilms tumor (Wegert et al., 2015) and of hFK (O'Brien et al., 2016).

To determine the effect on sequence specificity of the Q177R variant, we expressed the SIX1 (reference allele) and SIX1-Q177R DNA-binding homeodomains *in vitro* and assayed their specificities in parallel by protein binding microarrays (PBMs) (Table S2) (Berger et al., 2006). PBMs have been shown to reliably reflect *in vivo* binding preferences of transcription factors, such that the PBM-derived primary binding motif generally constitutes all or a main portion of the *in vivo*-derived motif (Wei et al., 2010; Fang et al., 2012). The primary and secondary motifs (Badis et al., 2009) recognized by both alleles are shown in Fig. 3A; a replicate experiment on an independent array yielded qualitatively similar sequence logos. The human SIX1 reference allele primary motif ‘GGGTATCA’ matches that of mouse Six1 in the UniProbe Database (Hume et al., 2014). It also closely matches the left half of the hFK SIX1 motif, although less so when compared to the SIX1-Q177R tumor motif (Fig. 3A,B). As the PBM assays use only homeodomain protein fragments, these differences between the PBM-derived and *in vivo* ChIP-seq-derived motifs could be influenced by interactions governed by amino acid residues outside of the homeodomain, as well as protein co-factors and the chromatin context *in vivo.* For example, a thorough examination of full-length SIX1 binding preferences by using electrophoretic mobility shift assays (EMSAs) found that the suffix ‘C’ nucleotide in the *in vivo*-derived motifs (Fig. 3B) is necessary for high affinity binding *in vitro*. However, a probe containing the PBM-derived motif in addition to the necessary suffix was bound most strongly (Liu et al., 2012).

Remarkably though, the Q177R allele primary motif very closely matches the Q177R tumor motif, with a strong ‘GTGT’ preference (Fig. 3A,B). To quantify the binding of each allele to each motif, we calculated ‘pattern E-scores’ (see Materials and Methods) for each replicate of each protein to the four patterns shown in Fig. 3A. The Q177R allele showed dramatically reduced binding to the SIX1 reference primary motif and essentially no binding to the reference secondary motif, while the alternate motifs bound by the Q177R allele showed similarly poor binding by the SIX1 reference allele (Fig. 3A). Crucially, while the primary motif preference is altered by the Q177R variant, these data indicate that both proteins can still bind to the preferred motif of the other. Overall, our findings validate those described by Wegert et al. (2015) from primary Wilms tumor tissue and confirm that the distinct binding specificity of the mutant protein is a direct result of the Q177R mutation.

### Core NPC regulatory target genes of SIX1 in hFK are conserved in Wilms tumor and several differentially expressed genes in SIX1/2-Q177R tumors represent putative regulatory targets of SIX1-Q177R

**Fig. 4.**
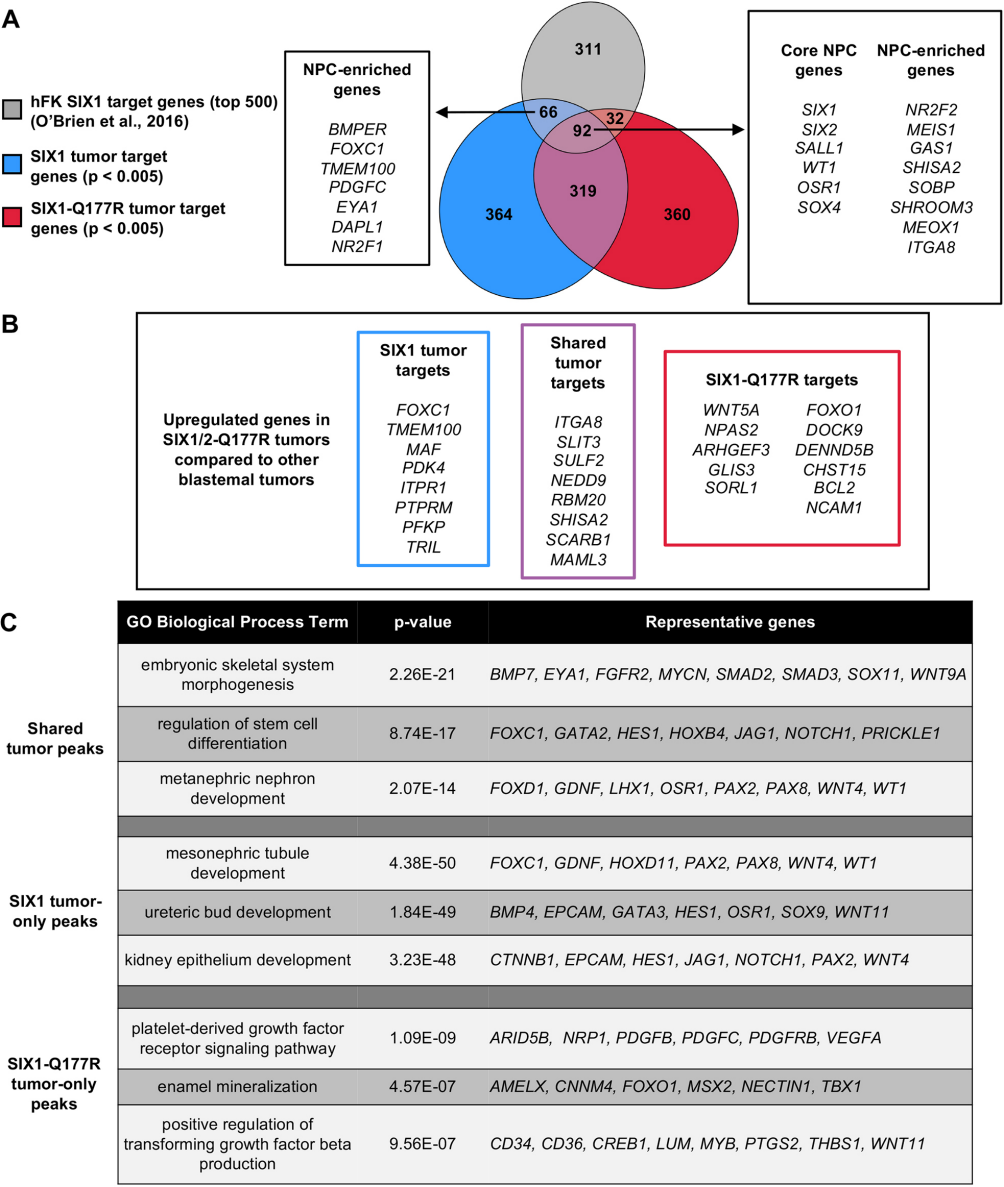
**Core NPC regulatory target genes of SIX1 in hFK are conserved in Wilms tumor and differentially expressed genes in SIX1/2-Q177R tumors represent putative regulatory targets of SIX1-Q177R.** (A) Venn diagram displaying overlap of the top 500 SIX1 target genes in hFK (O'Brien et al., 2016), with target genes of SIX1 and SIX1-Q177R in Wilms tumor identified using GREAT (see Materials and Methods). Shown are core NPC genes and other known NPC-enriched genes that were identified as putative targets in the indicated sample overlaps. (B) Putative target genes of SIX1 and/or SIX1-Q177R in Wilms tumor that were also found to be significantly upregulated in SIX1/2-Q177R Wilms tumors (*P*<0.005). (C) Selected significantly enriched biological processes and representative putative target genes that are associated with shared tumor, SIX1 tumor-only and SIX1-Q177R tumor-only ChIP-seq peaks generated using GREAT.

To identify putative target genes of SIX1-Q177R in Wilms tumor and characterize these as either tumor-specific or conserved normal kidney regulatory targets, we integrated three SIX1 ChIP-seq peak sets from SIX1 tumor, SIX1-Q177R tumor and from hFK (Wegert et al., 2015; O'Brien et al., 2016). The Genomic Regions Enrichment of Annotations Tool (GREAT) (McLean et al., 2010) was used to identify potential target genes regulated by SIX1 and/or SIX1-Q177R in Wilms tumor by ChIP-seq peak-gene association (*P*<0.005, see Materials and Methods). Putative target genes in Wilms tumor were compared to the top 500 target genes of SIX1 in hFK (O'Brien et al., 2016). Though 18% of the top 500 target genes in hFK were also identified in both tumor datasets, these shared targets included almost 50% of the top 100 target genes in hFK (44%) (Table S3). Notably, these shared target genes included several core NPC target genes that have previously been identified as being shared regulatory targets of the NPC transcription factors Six2, Hoxd11, Wt1 and Osr1 in mouse (O'Brien et al., 2018a), i.e. *SIX1, SIX2*, *SALL1*, *WT1*, *OSR1* and *SOX4* (Fig. 4A).

In addition, several established NPC-enriched genes had been identified in all three datasets including *SHISA2* and *ITGA8*, both of which were found to be upregulated in SIX1/2-Q177R tumors (Figs 4A and 1C) (O'Brien et al., 2018a; Müller et al., 1997; Lindström et al., 2018b). Genes identified as targets of wild-type SIX1 in both Wilms tumor and hFK but not of SIX1-Q177R include established NPC-enriched genes *FOXC1* and *TMEM100*, both of which were also found to be upregulated in SIX1/2-Q177R tumors (Figs 4A and 1C). Considering that SIX1/2-Q177R is primarily heterozygous in Wilms tumors, the upregulation of some targets associated with this mutation might result from a synergistic interaction between wild-type and mutant alleles. Comparison of putative target genes with other upregulated genes identified in our DGE analyses revealed many potential regulatory targets of SIX1-Q177R, either shared with SIX1 or, possibly, exclusive to SIX1-Q177R, including *WNT5A* (Fig. 4B). This analysis demonstrates that both SIX1 and SIX1-Q177R recapitulate key NPC regulatory activities that could contribute to the persistence of NPC-like characteristics in Wilms tumor, and that SIX1-Q177R is a candidate for regulation of several differentially expressed genes in SIX1/2-Q177R tumors.

### Binding sites exclusive to SIX1-Q177R in Wilms tumor are associated with genes enriched in distinct biological processes

To more broadly characterize the potential regulatory activities of SIX1 and SIX1-Q177R in Wilms tumors, GO analysis was performed by using GREAT with the following motif-enriched Wilms tumor ChIP-seq peak sets: peaks exclusive to the SIX1-Q177R dataset (9483 peaks), peaks exclusive to the SIX1 dataset (9759 peaks), and peaks shared by both SIX1-Q177R and SIX1 datasets (7984 peaks) (Table S3). Consistent with our observations shown in Fig. 4A, shared peaks and SIX1-only peaks were mostly associated with genes enriched in biological processes related to kidney development. By contrast, no biological process explicitly related to kidney development was found to be significantly enriched in the SIX1-Q177R-only peak dataset (summarized in Fig. 4C, additional GO terms shown in Fig. S2A). These distinct enriched processes included regulation of signaling pathways, such as ‘platelet-derived growth factor receptor signaling pathway’ and ‘positive regulation of transforming growth factor beta production’ and might reflect true broader regulatory consequences downstream of SIX1-Q177R in Wilms tumors. However, we did not find evidence of differential expression of genes related to these processes in our DGE analyses (Table S1). Furthermore, the SIX1-Q177R tumor that was used to generate the ChIP-seq data was homozygous for the mutation, while the overwhelming majority of SIX1/2-Q177R tumors in the microarray and RNA-seq datasets harbored heterozygous mutations (Wegert et al., 2015; Walz et al., 2015). Therefore, binding sites unique to the SIX1-Q177R tumor and associated with genes for which there was no evidence of differential expression could be indicative of regulatory activity of the mutant protein unique to the homozygous state.

### *WNT5A* represents a putative regulatory target of both SIX1 and SIX1-Q177R in Wilms tumor

**Fig. 5.**
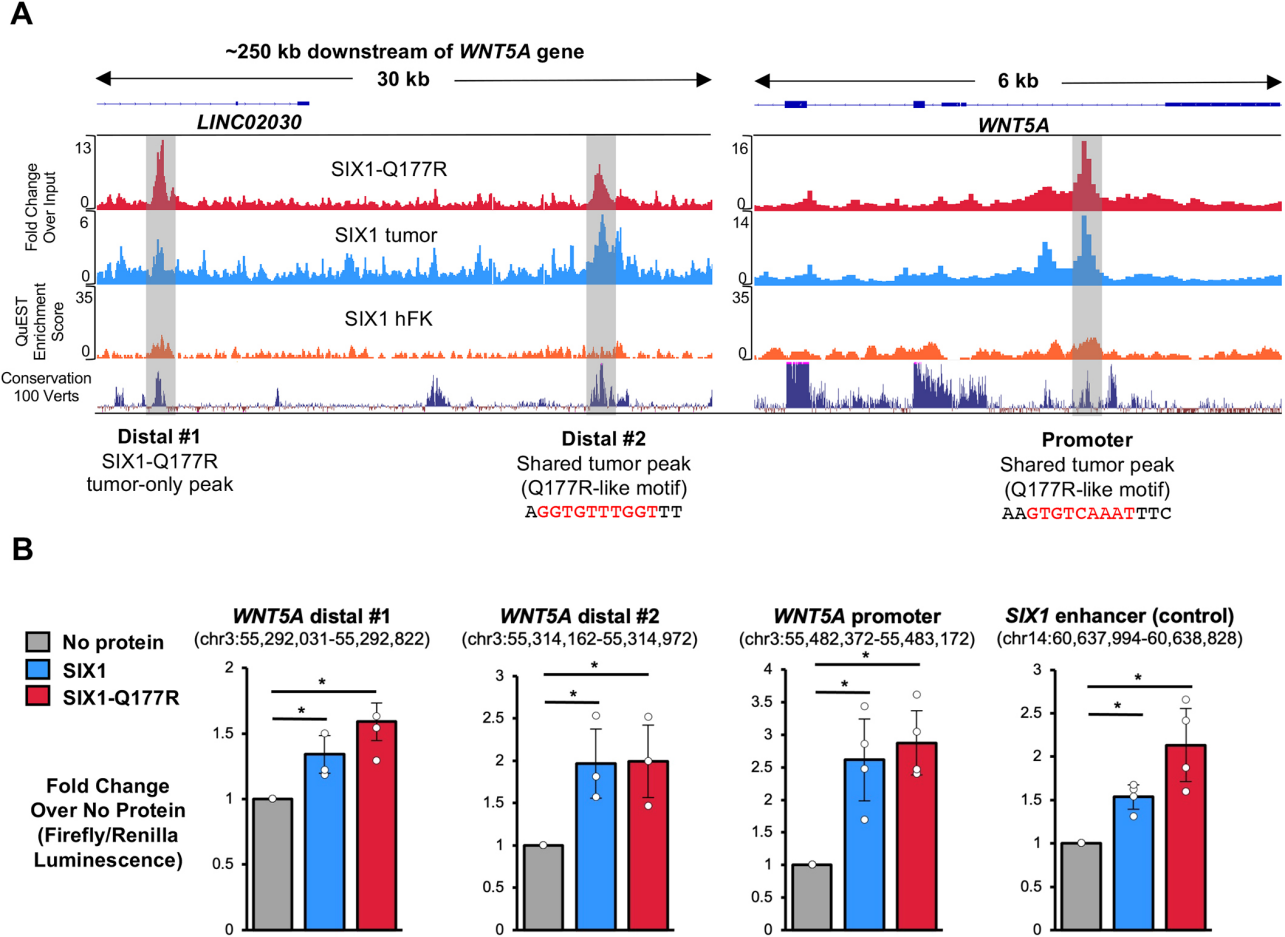
**Conserved promoter and putative distal cis-regulatory elements (CREs) of *WNT5A* bound by SIX1 and SIX1-Q177R in Wilms tumor show enhanced expression in *in vitro* luciferase assays with either protein.** (A) Top: IGV genome browser snapshots displaying SIX1-Q177R tumor (red), SIX1 tumor (blue) and hFK SIX1 (orange) ChIP-seq tracks. Bottom: genome sequence conservation track of 100 vertebrates sequence obtained from the UCSC genome browser. Regions underlaid in gray indicate positions of SIX1 and/or SIX1-Q177R motif-enriched peaks in Wilms tumors. (B) Bar graphs displaying results of the luciferase enhancer assays for each of the indicated DNA elements. Bar height represents the mean, error bars indicate s.d. *P<0.05 (one-tail Student's *t*-test), *n*≥3 biological replicates per DNA element.

In addition to a site bound only by SIX1-Q177R, Wilms tumor ChIP-seq peaks containing SIX1/SIX1-Q177R motifs bound by both SIX1 and SIX1-Q177R, were identified at proximal and distal regions near the *WNT5A* locus. These were not called as peaks in the hFK dataset (Fig. 5A) (O'Brien et al., 2016). Significant DNA sequence conservation suggests that the distal sites represent CREs. Interestingly, both shared peaks contained DNA sequences that are highly congruent with the primary SIX1-Q177R-binding motifs identified in the ChIP-seq and PBM datasets (Fig. 3A,B) (Wegert et al., 2015). Similar examples of shared SIX1/SIX1-Q177R peaks containing the preferred SIX1-Q177R motif were found at putative CREs for upregulated WNT signaling effectors identified in our DGE analysis, including *FZD4, FZD6*, *CCDC88C*, *KREMEN1* and *SLIT3* (Fig. S2B). The shared peak within an intron of *WNT5A* is within a region that has previously been characterized to be a *WNT5A* promoter (Katula et al., 2012; Danielson et al., 1995). Intriguingly, this shared peak overlaps with a Six1 ChIP-seq peak identified in developing mouse cochlea, indicative of an evolutionarily conserved Six1-binding site (Li et al., 2020). Additionally, immunostaining of E10.5 mouse embryo sections revealed colocalization of Six1 and Wnt5a within the presumptive myotome, lending further support to the potential for *Wnt5a/WNT5A* regulation by Six1/SIX1 *in vivo* (Fig. S2C).

Hi-C chromatin interaction data generated as part of the ENCODE consortium illustrate that the *WNT5A* locus, as well as regions comprising the putative distal CREs, is located within a topologically associating domain (TAD) in several cell lines, including: HepG2, IMR-90, MG63 and THP-1 (Fig. S2D) (Wang et al., 2018; Moore et al., 2020; Rao et al., 2014; Xu et al., 2022; Phanstiel et al., 2017). TADs are regions of the genome that are predominantly unchanged between cell types and defined by frequent intrachromosomal contacts, such as enhancer–gene interactions, and infrequent contacts with regions outside of the TAD (reviewed by Dixon et al. 2016). Moreover, a recent study has identified a chromosomal compartment encompassing this region in which local DNA interactions become more frequent within a cell line model of prostate cancer progression that results in enhanced expression of *WNT5A,* highlighting the importance of gene regulatory interactions within this TAD in the regulation of *WNT5A* expression (Martin et al., 2022). We then explored the potential regulation of gene expression by SIX1 and SIX1-Q177R through the putative *WNT5A* CREs. For this, we used MCF-7 cells transiently transfected with the established SIX1 co-factor EYA1 (Li et al., 2003) together with either SIX1 or SIX1-Q177R overexpression constructs and measured luciferase activity from minimal promoter constructs containing each putative regulatory element. An established enhancer for *SIX1* was utilized as a control (O'Brien et al., 2016). As shown in Fig. 5B, both wild-type and mutant SIX1 drove significant (and similar) expression levels of luciferase from the promoter DNA element as well as both distal DNA elements when compared with the SIX1 enhancer control. These data support the novel regulation of *WNT5A* expression by both wild type SIX1 and SIX1-Q177R.

### SIX1-Q177R binds the *WNT5A* promoter element with higher affinity than SIX1

**Fig. 6.**
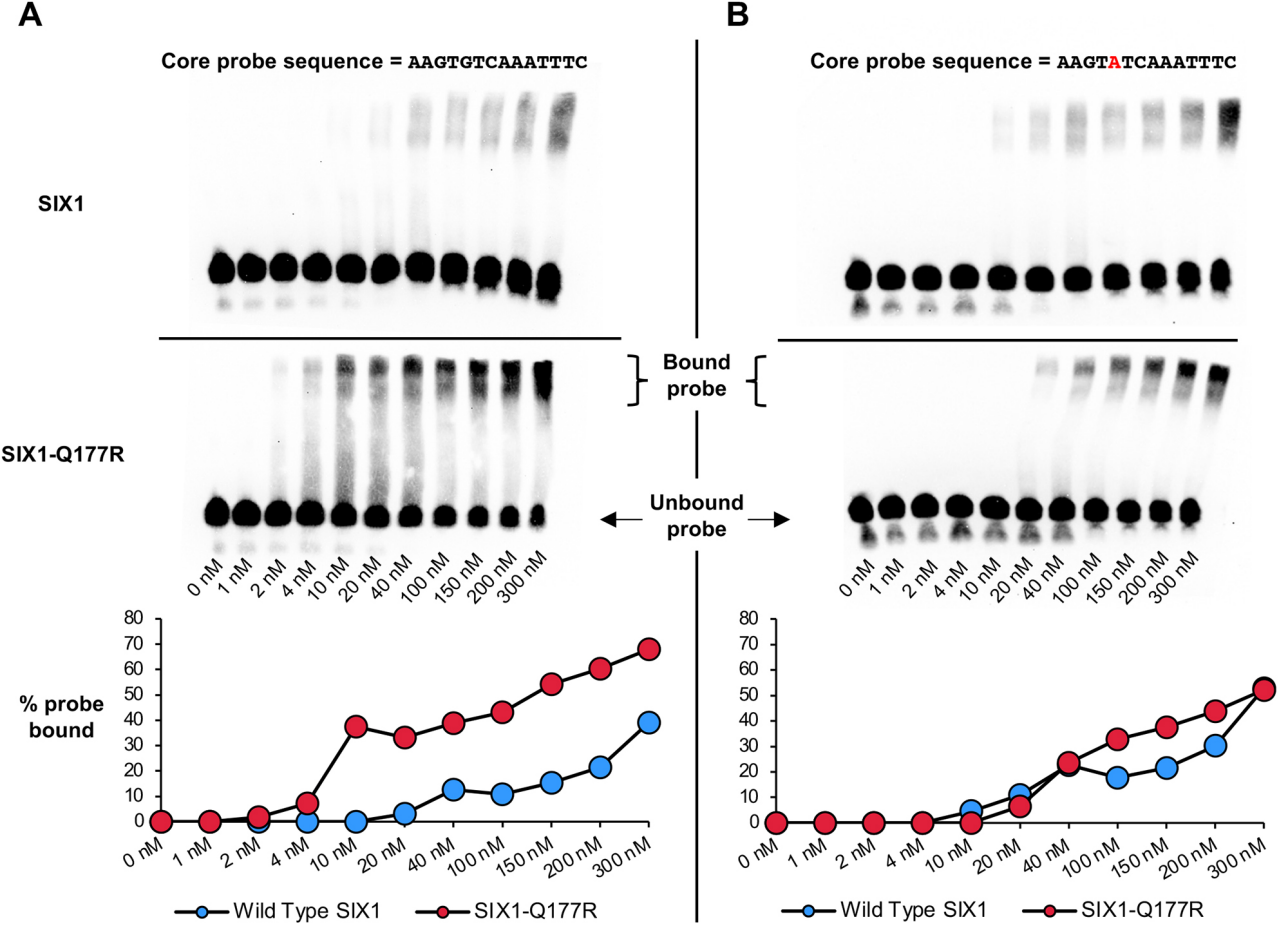
**SIX1-Q177R binds the core DNA motif sequence within the *WNT5A* promoter ChIP-seq peak with a higher affinity than SIX1.** (A,B) Top: chemiluminescence EMSA images of purified recombinant SIX1 and SIX1-Q177R protein at concentrations as indicated, together with biotin-labeled oligonucleotide probes containing the core probe DNA motif sequences (shown above), derived from wild-type *WNT5A* promoter ChIP-seq peak (A) and mutated *WNT5A* promoter ChIP-seq peak (B). Bottom: quantification of EMSA-derived DNA-protein binding data determined from signal intensities of bound and unbound probe by using ImageJ software.

The SIX1-Q177R mutation is almost exclusively heterozygous in Wilms tumors and, in the heterozygous context, both wild-type *SIX1* and *SIX1-Q177R* alleles are expressed at similar levels (Walz et al., 2015; Wegert et al., 2015). Accordingly, differences in binding affinity at binding sites shared between the two proteins might contribute to aberrant gene expression *in vivo*. To investigate this possibility, we carried out EMSAs using purified recombinant wild-type SIX1 or SIX1-Q177R full-length protein and biotinylated oligonucleotide probes containing the putative DNA-binding sequence found within the *WNT5A* promoter peak, which is highly congruent with the preferred SIX1-Q177R motif discovered in the ChIP-seq and PBM datasets (Fig. 3A,B) (Wegert et al., 2015). As shown in Fig. 6, SIX1-Q177R binds this sequence with an affinity that is ∼10-fold higher compared to that of SIX1. Furthermore, changing guanine to adenine at the nucleotide position that is flanked by thymines, such that the motif now resembles the SIX1-preferred motif (Fig. 3A,B) (Wegert et al., 2015), resulted in a loss of affinity of SIX1-Q177R with a concomitant, albeit modest increase in affinity of SIX1. That SIX1-Q177R binds the ‘mutated’ probe in which the DNA sequence aligns with the wild-type SIX1 motifs from the ChIPseq and PBMs with similar affinity to SIX1 is somewhat contradictory to the PBM results shown in Fig. 3A, in which the Q177R homeodomain bound this motif with seemingly reduced affinity compared to that of the wild-type homeodomain (O'Brien et al., 2016; Wegert et al., 2015). However, while protein homeodomain fragments were used in PBMs, full-length proteins were used in the EMSAs. As such, intramolecular interactions within the tertiary structure of full-length peptides might contribute to binding of less-preferred DNA motifs. Our data suggest that, under conditions of equal expression of SIX1 and SIX1-Q177R, the enhanced affinity of SIX1-Q177R promotes preferential binding to the *WNT5A* promoter over the wild-type protein, plausibly facilitating elevated expression of *WNT5A*.

## DISCUSSION

WNT5A is a potent and conserved secreted signaling molecule known for its role in modulating morphogenesis and tissue movements through non-canonical WNT/planar cell polarity (PCP) signaling (Moon et al., 1993; Qian et al., 2007; Nishita et al., 2010). Though it has been extensively studied in cancer and may both activate or inhibit cancer progression dependent on tissue context (reviewed by McDonald and Silver, 2009), investigations of WNT5A in Wilms tumor are scarce. An earlier analysis of *WNT5A* expression in FHWT Wilms tumors has demonstrated a positive correlation between low *WNT5A* expression (compared to expression in hFK control) and blastemal predominant histology (Tamimi et al., 2008). While we do not directly compare the magnitude of *WNT5A* expression in Wilms tumors to that in hFK, the results of our DGE analysis appear to generally agree with the findings by Tamimi and colleagues, except in the case of SIX1/2-Q177R tumors. *WNT5A* expression was lower in blastemal tumors compared to that in mixed/epithelial/stromal histology tumors (log_2_-fold change=−1.45, adjusted *P*-value=0.002, Table S1), while expression in SIX1/2-Q177R tumors was upregulated compared to that in other blastemal tumors – albeit not significantly different to that in mixed/epithelial/stromal tumors. With *WNT5A* being one of only seven genes significantly upregulated in SIX1/2-Q177R tumors compared to other blastemal tumors in both expression datasets (Fig. 1A), it is a strong candidate for promoting tumor progression and the chemotherapeutic resistance associated with SIX1/2-Q177R. Beyond the non-canonical WNT signaling pathway, WNT5A has been shown to enhance the half-life of p53, supporting a slow-cycling state that is thought to contribute to therapeutic resistance in melanoma (Webster et al., 2020). Notably, mutations in *TP53* have mostly been associated with diffuse anaplastic Wilms tumor and only rarely present in FHWT (Gadd et al., 2017). Accordingly, *TP53* was expressed in all tumors without evidence of differential expression in our DGE analyses (Table S1). Considering the relevance of WNT5A in other cancers, our findings suggest that further investigations of WNT5A in Wilms tumor, and its potential as a therapeutic target, are warranted.

In the context of kidney development – though *Wnt5a* expression is absent beyond E11.5 in mouse NPCs and *WNT5A* is not expressed significantly in human NPCs (Fig. 1C) – this cell population is likely to remain capable of responding to Wnt5a/WNT5A ligand through the expression of appropriate receptors. Ror2 is a tyrosine kinase-like orphan nuclear receptor responsive to Wnt5a and is expressed from E10.5-E15.5 in the MM (Nishita et al., 2014; Hikasa et al., 2002; Oishi et al., 2003; Schambony and Wedlich, 2007; Mikels et al., 2009). Many candidate WNT5A receptors are expressed in hFK as well as in Wilms tumor based on the RNA-seq datasets used in this study. This includes *ROR2*, *RYK*, *MCAM*, *FZD2*, *FZD3*, *FZD4*, *FZD6* and *FZD7* (Fig. S3) (reviewed by Kumawat and Gosens, 2016). Interestingly, an early study co-culturing isolated MM with different WNT ligand-expressing cells has demonstrated that – in contrast to other Wnt ligands (i.e. Wnt1a, Wnt3a, Wnt4, Wnt7a and Wnt7b) – *Wnt5a*-expressing cells do not induce tubulogenesis of the MM (Kispert et al., 1998), suggesting Wnt5a-mediated non-canonical WNT signaling does not promote differentiation in the NPC niche. While the possible effects of *WNT5A* expression in NPCs *in vivo* is unclear, the developmental roles of other WNT ligands are more established and may provide clues as to the oncogenic potential of upregulated *WNT5A* within this niche.

Both canonical and non-canonical WNT signaling are required for proper patterning and development of the murine kidney. Wnt9b is secreted from the ureteric epithelium and signals through canonical WNT/β-catenin-mediated transcriptional regulation, with low levels of Wnt9b promoting self-renewal of NPCs and higher levels promoting induction of differentiation (Carroll et al., 2005; Ramalingam et al., 2018; Karner et al., 2011; Park et al., 2007). Wnt4 is thought to act through a non-canonical mechanism by modulating intracellular Ca^2+^ levels, and is required for mesenchymal-to-epithelial transition and the formation of the renal vesicle (Stark et al., 1994; Kispert et al., 1998; Tanigawa et al., 2011). The ureteric epithelium is also a source of the non-canonical ligand Wnt11 that is required for proper polarity of NPCs within the cap mesenchyme, thereby maintaining an organized niche and ensuring sufficient nephron endowment (Majumdar et al., 2003; O'Brien et al., 2018b). *Wnt11* knockouts displayed dispersion of NPCs within the normally tightly packed cap niche as well as accelerated differentiation of the NPCs. Strikingly, this phenotype did not manifest in significant changes in the transcriptome of NPCs in *Wnt11* knockout kidneys, illustrating the importance of cell–cell interactions and non-transcriptional mechanisms in NPC maintenance (O'Brien et al., 2018b). Taken together, a delicate interplay between non-canonical and canonical WNT signaling governs the fate of NPCs in the cap niche. Mounting evidence points to imbalances in canonical WNT/β-catenin pathway signaling as a central mechanism driving Wilms tumorigenesis, particularly in cases of WT1-inactivating and CTNNB1-activating mutations (reviewed by Perotti et al., 2013; Fukuzawa et al., 2009; Li et al., 2004). Thus, our observations suggest a similar result can be achieved in SIX1/2-Q177R mutant tumors through modulation of different upstream effectors. Altogether, this network of factors may support non-canonical mechanisms that perturb the differentiation capacity of these cells, and promote tumor formation and growth.

The finding that some podocyte-associated genes including *WNT5A* are specifically upregulated in SIX1/2-Q177R blastemal tumors, and that the canonical podocyte lineage-specific markers MAFB and PODXL colocalize with SIX1 and WNT5A in Wilms tumor tissue is intriguing when considered within the framework of the NPC differentiation continuum. Recently, time-lapse imaging of the developing mouse kidney has demonstrated that the last committed NPCs integrating into the renal vesicle contribute to the podocyte lineage (Lindström et al., 2018a). Pseudo-time temporal analysis of single-cell RNA-seq data from nephrogenic hFK tissue reinforced those findings, indicating podocytes are the most closely related descendent of NPCs and arise from a distinct differentiation timeline to that of the tubule precursors, so much so that the authors concluded podocytes differentiate directly from committed NPCs (Lindström et al., 2018a). From a signaling standpoint, canonical WNT/β-catenin signaling has been demonstrated to inhibit podocyte differentiation in mouse and chick model systems, as well as in human induced pluripotent stem cell-derived podocytes (Lindström et al., 2015; Grinstein et al., 2013; Yoshimura et al., 2019). Thus, NPC activation of early podocyte transcriptional programs may represent the differentiation path of least resistance, and perturbation of canonical WNT signaling in favor of non-canonical WNT signaling could further encourage this progression.

As opposed to the upregulation of *SIX2*/*PAX2*/*SALL1* in chemotherapy-treated blastemal tumors, our DGE analysis revealed no evidence for significant differential expression of core NPC transcription factors when comparing chemotherapy-naïve blastemal and mixed/epithelial/stromal tumors. This supports the characterization of post-chemotherapy Wilms tumor blastema as more NPC-like transcriptionally compared to other histology but indicates this same characterization of chemotherapy-naïve blastema is inconsistent. Accordingly, COG guidelines do not stratify tumors by blastemal/stromal/mixed histology prior to chemotherapy (Vujanic et al., 2022). Thus, chemotherapy-naïve histology might not be representative of the intrinsic transcriptional state, rather it might more reflect niche differences in extracellular matrix composition and cell-cell interactions. In both datasets, core NPC genes were also not found to be significantly differentially expressed in SIX1/2-Q177R tumors compared to other blastemal tumors. However, our ChIP-seq analysis identified core NPC genes to be potential regulatory targets shared with SIX1 in hFK. This suggests that SIX1/2-Q177R does not exert its regulatory effect through enhancement of the core NPC transcriptional network but aids in sustaining this network of genes in addition to the potential role in modulation of the WNT signaling pathway as described in this study.

Last, our PBM and EMSA data confirm the altered DNA-binding motif preference of SIX1-Q177R to be a direct consequence of this specific mutation and indicate the mutant protein binds its preferred motif with an affinity that is greater than that of wild-type SIX1 for its preferred motif. In agreement with the Wilms tumor ChIP-seq data, these PBM and EMSA data also demonstrated that both proteins can bind the other's preferred motif both *in vitro* and *in vivo*, although our EMSA data suggest that SIX1-Q177R binds the SIX1-preferred motif with similar affinity to that of SIX1. Transcription factor binding affinity can positively correlate with both timing and magnitude of target gene expression (Gao and Stock, 2015; Sharon et al., 2012). Therefore, the seemingly preserved affinity of SIX1-Q177R for the wild-type SIX1 motif, in addition to enhanced affinity for a divergent motif, could have significant consequences within developmental gene regulatory networks. Despite the evidence presented here for enhanced binding affinity of SIX1-Q177R and significant association of this mutation with the upregulation of WNT signaling pathway effector genes, SIX1/2-Q177R is unlikely to be solely responsible for this DGE in Wilms tumors. Interactions with transcriptional co-factors and/or cooperation with wild-type SIX1/2 or with other common Wilms tumor-associated mutations – such as the *DGCR8*/*DROSHA* mutations mentioned earlier, or loss of imprinting/loss of heterozygosity at chromosome 11p15 resulting in the overexpression of *IGF2* (affecting an estimated 75–80% of FHWT and blastemal tumors) (Walz et al., 2015; Wegert et al., 2015) – might be necessary to enhance the regulatory effect of SIX1/2-Q177R and will require further investigation.

Ultimately, our studies are limited by the availability of Wilms tumor tissues – particularly blastemal predominant tissue and SIX1/2-Q177R mutant tumors – for thorough experimental validation. In addition, the homozygosity of the SIX1-Q177R mutation in the tumor from which the ChIP-seq had been derived as well as the differing treatment regimens between the tumors that had been included in the ChIP-seq and RNA-seq datasets, make for imperfect comparisons. Furthermore, the bulk gene expression datasets do not offer the resolution of a single-cell analysis to assign gene expression signatures to distinct cell populations and characterize the degree of intratumor heterogeneity at the transcriptome level. Nevertheless, these genomic datasets from limited tissue sources, such as Wilms tumor, are invaluable resources containing crucial information on the molecular mechanisms intrinsic to these tumors. Through novel integrated analyses of these datasets, we uncovered an enhanced gene expression signature that is associated with the WNT signaling pathway in chemotherapy-naïve tumors harboring the relapse-associated SIX1/2-Q177R mutation, including the gene encoding for the non-canonical WNT ligand *WNT5A* in both chemotherapy-naïve and chemotherapy-treated tumors, thereby linking this signaling pathway to both the maintenance and recurrence of these tumors. Our analysis of SIX1- and SIX1-Q177R-binding data from Wilms tumors, and our *in vitro* validation of binding-affinity differences between wild-type and mutant protein – particularly at a promoter element for *WNT5A* – implicates the enhanced binding affinity of SIX1/2-Q177R at putative CREs in the regulation of expression of these differentially expressed genes. Overall, our findings have uncovered disrupted signaling networks linked to the regulatory behavior of SIX1/2-Q177R, providing additional clues to aid in our understanding of the complex underlying biology of Wilms tumors.

## MATERIALS AND METHODS

### Cell culture and transfections

HEK293T [obtained from University of North Carolina (UNC) Lineberger Comprehensive Cancer Center Tissue Culture Facility] and MCF-7 (gift from Dr Richard Cheney, UNC-Chapel Hill) cell lines were cultured in DMEM/F12 w/ L-glutamine and HEPES (Gibco)+10% fetal bovine serum (Omega Scientific)+1× penicillin/streptomycin (Gibco), unless indicated otherwise. Medium was exchanged every 3–4 days and cells were passaged at confluence. Transfections for all assays were carried out using the Lipofectamine 3000 kit (Invitrogen) following manufacturer's protocol, scaled for the tissue culture vessel used. Cells were assayed ∼48 h post transfection.

### General cloning

All restriction digest reactions were carried out at 37°C using CutSmart Buffer (New England Biolabs). All restriction enzymes were from New England Biolabs, unless indicated otherwise. Restriction digest products were subjected to gel electrophoresis in 1% agarose gel+1× SYBR Safe DNA Gel Stain (Invitrogen). DNA bands of digested DNA were excised and purified using NucleoSpin Gel and PCR Clean-Up kit (Machery-Nagel) following the manufacturer's protocol. All ligations were carried out at room temperature (RT) using T4 DNA ligase (New England Biolabs) following the manufacturer's protocol. Unless indicated otherwise, plasmids were transformed in 5-alpha competent *E. coli* cells (New England Biolabs) following the manufacturer's protocol. LB agar plates containing 1× ampicillin (Sigma-Aldrich) were then streaked with transformed bacteria and incubated at 37°C overnight. Individual bacteria colonies were picked using P1000 pipet tips and cultured in LB broth+1× ampicillin at 37°C on orbital shaker from several hours to overnight. Plasmids were purified from cultures using NucleoSpin Plasmid EasyPure purification kit (Machery-Nagel) following manufacturer's protocol. Plasmids were submitted to GENEWIZ (Azenta Life Sciences) for Sanger sequencing to verify DNA sequence. Purified plasmids verified by DNA sequencing were either used directly in applicable assay or re-transformed in 5-alpha cells; an individual bacteria colony was used to inoculate a 200 ml liquid culture in LB broth+1× ampicillin with incubation overnight at 37°C on an orbital shaker. Plasmids were purified using the NucleoBond Xtra Midi Plus EF Kit (Machery-Nagel) following the manufacturer's protocol, including the use of NucleoBond finalizers.

### SDS-PAGE and western blotting

All samples were prepared using LDS sample buffer (Invitrogen)+2.5% 2-mercaptoethanol (Sigma-Aldrich) and incubated at 70°C for 10 min prior to electrophoresis. Samples were run on Novex 4–20% Tris-glycine gels (Invitrogen) together with Precision Plus Protein All Blue Prestained Protein Standards (Bio-Rad), using 1× Tris-glycine running buffer (25 mM Tris base (Fisher BioReagents), 190 mM glycine (Fisher Chemical) and 3.5 mM SDS (Fisher Chemical) in a Mini Gel Tank (Invitrogen). Proteins were then transferred to a nitrocellulose membrane (GE Water and Process Technologies) using a wet transfer protocol and a Mini Trans-Blot Cell (Bio-Rad) transfer apparatus at 4°C, 100V for 1 h in transfer buffer (192 mM glycine, 25 mM Tris base, 20% methanol). Membranes were then washed several times with double-distilled H_2_O. Membranes were blocked in PBS+0.1% TWEEN-20 (Fisher BioReagents)+5% dry milk at RT for 20–40 min and residual milk was washed from membrane with H_2_O. Primary antibody solutions were made in PBS+0.1% TWEEN-20+3% BSA (Fisher BioReagents). Unless indicated otherwise, membranes were incubated in primary antibody solution with gentle rocking at either RT for 1 h or overnight at 4°C. Membranes were washed at least three times with PBS+0.1% TWEEN-20, 5 min per wash. Secondary antibody solutions were made in PBS+0.1% TWEEN-20+1% dry milk. Membranes were incubated in secondary antibody solution for 1 h at RT. Membranes were washed at least three times with PBS+0.1% TWEEN-20 and then incubated in Pierce ECL Western Blotting Substrate (Thermo Fisher Scientific) for 1–2 min at RT. Blots were imaged using the iBright FL1500 Imaging System (Invitrogen).

### ChIP-seq data analysis

Week 17 human fetal kidney SIX1 ChIPseq data were deposited in the Gene Expression Omnibus (GEO) data repository under accession number GSE73867 (O'Brien et al., 2016). Pre-processed SIX1 ChIP-seq data from Wilms tumors were kindly shared by Dr Manfred Gessler (Wegert et al., 2015) in the form of bigWig files from two SIX1 wild-type ChIP-seq replicates (one replicate from each of the two tumors) and two SIX1-Q177R ChIP-seq replicates (each from the same tumor) generated using deepTools bamCompare (Ramírez et al., 2016) [50 bp bins, normalized to pooled input by signal extraction scaling (SES) (Diaz et al., 2012)]. BigWig files were converted to bedGraph format using UCSC tools bigWigToBedGraph (Kent et al., 2010). Log_2_-fold enrichments over input were averaged between each pair of replicates, generating a single bedGraph file for each. Genome coordinates were transformed from hg19 genome assembly to hg38 using the UCSC LiftOver tool (Kuhn et al., 2012). Peaks were called using MACS2 bdgpeakcall (Zhang et al., 2008) through the Galaxy web platform (https://usegalaxy.org/) (Afgan et al., 2018) using a log_2_-fold cut-off of±2. Peak overlaps between samples were identified by using bedtools (Quinlan and Hall, 2010), intersect intervals in Galaxy. Peak sequences were obtained by using bedtools GetFastaBed in Galaxy and these FASTA files were used as input for motif discovery in STREME (MEME Suite 5.4.1) (Bailey et al., 2015; Bailey, 2021) using default settings. The identified SIX1 or SIX1-Q177R motifs from STREME were also used as input into FIMO (Grant et al., 2011) using a *P*-value cut-off of <1E^−3^. The peak coordinates containing the desired motifs were converted to BED format and duplicate coordinates within each file were removed using bedtools MergeBED in Galaxy. These BED files were then used as input in the Genomic Regions Enrichment of Annotations Tool (GREAT; https://great.stanford.edu/great/public/html/) (McLean et al., 2010) using whole genome background and basal plus extension association rules, changing distal up to 500 kb. Putative target genes were identified by using a binomial *P*-value cut-off of *P*<0.005.

### Protein-binding microarrays

#### Cloning

DNA sequences flanked by restriction sites XhoI and NdeI, and encoding for N-terminal GST-tagged-SIX1 or N-terminal GST-tagged-SIX1-Q177R homeodomains were synthesized by Integrative DNA Technologies (IDT) as gBlocks (Table S5). PCR using Q5 High-Fidelity DNA Polymerase (New England Biolabs) was used to amplify gBlocks and PCR products were purified using the Nucleospin Gel and PCR Cleanup kit following the manufacturer's protocol. DHFR control plasmid from PURExpress In Vitro Protein Synthesis Kit (New England Biolabs) was used as backbone for subsequent ligations. DHFR control plasmid and amplified gBlocks were digested with XhoI and NdeI restriction enzymes. Gel purification, ligation, transformation and subsequent purification was performed as described above under ‘General cloning’.

#### *In vitro* transcription and translation, and quantification of protein levels

*In vitro* transcription/translation was carried out using the PURExpress In Vitro Protein Synthesis kit following the manufacturer's protocol. 1 µl from each reaction was diluted 1:100 in nuclease-free H_2_O. 7 µl of each dilution was then used for SDS-PAGE alongside a dilution series of recombinant GST protein (Sigma-Aldrich #SRP5348) from 5 ng-200 ng. SDS-PAGE and western blotting were performed as described above, with the following changes: 90 min primary antibody incubation at RT (rabbit anti-GST, Sigma-Aldrich #G7781, 1:4000) and 30 min secondary incubation at RT (goat anti-rabbit HRP, 1:5000) (Fig. S4). Protein concentration was quantified using band intensities obtained by using the Gel tool in ImageJ (Schneider et al., 2012). A standard curve was generated using known recombinant GST bands in the gel. By using Microsoft Excel, a logarithmic line-of-best-fit was generated and used to quantify the mass of the *in vitro* transcribed/translated samples. Molarity of the purified protein samples was calculated using a molecular mass of 36.31 kDa, nuclease-free H_2_O was added to bring the molarity of each sample to 4.5 µM. Aliquots were collected and stored at −80°C.

#### Protein-binding microarrays

Protein binding microarrays (PBMs) were performed on universal ‘all 10-mer’ arrays in 8×60K format (GSE AMADID #030236, Agilent Technologies) essentially as described previously (Berger and Bulyk, 2009; Berger et al., 2006). PBM experiments were performed in duplicate at 300 nM final concentration of GST-tagged protein. Protein binding was detected with Alexa Fluor 488-conjugated anti-GST antibody (Invitrogen A-11001). Arrays were scanned using a GenePix 4400A microarray scanner (Molecular Devices). Raw data files were processed, and binding was quantified using the Universal PBM Analysis Suite (Berger and Bulyk, 2009). Motif position weight matrices were derived using the Seed-and-Wobble algorithm (Berger and Bulyk, 2009; Berger et al., 2006) and sequence logos were generated with enoLOGOS (http://www.benoslab.pitt.edu/cgi-bin/enologos/enologos.cgi) (Workman et al., 2005). Pattern E-scores were generated using the same algorithm and input files as 8-mer E-scores (Berger and Bulyk, 2009), with probes that contain matches to a given sequence pattern replacing probes containing a given 8-mer as the foreground in the calculation.

### Wilms tumor RNA-seq data analysis

Wilms tumor RNA-seq data were obtained through the National Cancer Institute TARGET Data Matrix (https://portal.gdc.cancer.gov/projects/TARGET-WT) in the form of gene quantification text files. From each gene quantification file, raw counts were transferred to an excel spreadsheet to create a count matrix. This count matrix was then used in Galaxy for differential gene expression (DGE) analysis using limma-voom with sample quality weights, filtering out genes expressed at low levels with counts per million<2 if the threshold was not met for all samples, and using a log_2_-fold change cut-off of ±1 and all other default settings (Smyth, 2005; Law et al., 2014; Liu et al., 2015). Volcano plots were made using the ‘Volcano Plot’ tool on the Galaxy web platform.

### Single-cell RNA-seq analysis

Raw and processed data were obtained from three studies: GSE112570, GSE139280, GSE124472 (only sample GSM3534656). All subsequent data processing and analyses were performed using the Seurat R package and following the analysis workflows outlined in vignettes ‘PBMC 3K guided tutorial’ and ‘Introduction to scRNA-seq integration’ (https://satijalab.org/seurat/index.html, Hao et al., 2021; Stuart et al., 2019; Butler et al., 2018; Satija et al., 2015). Briefly, each dataset was filtered by using the following parameters: LindströmWk14 – nFeature_RNA=1500–4000, mitochondrial counts<5%; LindströmWk17 – nFeature_RNA=1000–3500, mitochondrial counts<5%; TranWk17zone1 – nFeature_RNA=1000–5000, mitochondrial counts<5%. Each dataset was normalized independently; variable features were also identified independently. Integration features were selected and integration anchors identified. An integrated assay was then created, with data being scaled, and PCA and UMAP dimensional reduction performed using *n*=30 principal components/dimensions. Neighbors were found and clusters were found using resolution=1. Cluster markers were found using FindAllMarkers() function, min.pct=0.15, logfc.threshold=0.25, only.pos=TRUE. Cluster identities were assigned based on cluster annotations from the dataset source publications (Lindström et al., 2018a, 2021; Tran et al., 2019). Subset() function was used to remove cells annotated as proliferative NPCs or proliferative ICs. PCA and UMAP dimensional reduction, neighbor identification and cluster identification were then performed on the subset dataset same as above. AverageExpression() function was used to calculate the average expression of each gene in each cluster, used return.seurat=TRUE to return SeuratObject with scaled and centered expression values generated from ScaleData() function. Dot plots were generated using DotPlot() function.

### Luciferase assays

#### Cloning and plasmids

pBV-Luc was a gift from Bert Vogelstein (Addgene plasmid #16539; RRID:Addgene_16539). This plasmid encodes firefly luciferase driven by a minimal promoter element and was digested with NheI and HindIII; however, this digestion removed the minimal promoter from the pBV-Luc vector. SIX1_enhancer gBlocks (Table S5) were PCR amplified, digested with NheI and HindIII restriction enzymes and annealed with digested pBV-Luc. To re-insert the minimal promoter sequence, single-stranded DNA oligonucleotides containing the minimal promoter sequence flanked by 5′-HindIII and 3′-NcoI restriction sites (Table S5) were annealed in 1× annealing buffer [10 mM Tris base, 50 mM NaCl (Fisher Chemical), 1 mM EDTA (Invitrogen)] and incubated on a thermocycler at 95°C for 2 min followed by cooling to 25°C at a rate of −0.1°C/second. Annealed minimal promoter oligonucleotides and SIX1_enhancer-pBV-Luc were then digested with HindIII and NcoI restriction enzymes, and annealed. This plasmid was then used for all subsequent cloning of WNT5A proximal and distal CRE luciferase constructs using NheI/HindIII restriction sites (Table S5).

pRL-SV40P was a gift from Ron Prywes (Addgene plasmid #27163; RRID:Addgene_27163) and was used as *Renilla* luciferase expression control. Empty pCIG and empty pCS2+ plasmids were used as empty vectors for total DNA transfection normalization. The EYA1 overexpression plasmid was generated as follows: a sequence encoding EYA1-2xHA was generated by PCR amplifying *EYA1* coding sequence from pCS2+-EYA1-FLAG plasmid, swapping out FLAG tag for 2xHA tag (Table S5). pCS2+-EYA1-2xHA plasmid was generated using EcoRI and XbaI restriction sites in EYA1-2xHA fragment and pCS2+ plasmid. pCIG-SIX1 and pCIG-SIX1-Q177R plasmids were generated using gBlocks synthesized by Integrated DNA Technologies (IDT) containing the coding sequences for the respective proteins (Table S5). The gBlocks were PCR amplified and flanking ClaI and XhoI restriction sites were added and used for subsequent digestion and ligation into pCIG vector.

#### Luciferase assay

MCF-7 cells were cultured in 6-well plates to ∼90% confluence and medium was exchanged prior to transfection. For each biological replicate of each DNA element assayed, cells in three wells were transfected with 5 µg total DNA/well, yielding one ‘no protein control’ condition, one ‘SIX1/EYA1’ condition and one ‘SIX1-Q177R/EYA1’ condition. Control transfections consisted of 500 ng firefly luciferase vector, 10 ng *Renilla* luciferase vector, 1.5 µg empty pCIG and 3 µg empty pCS2+. Luciferase assays were performed using the Dual Luciferase Reporter Assay System (Promega). Cells in each well were harvested using a cell scraper and 100 µl 1× Passive Lysis Buffer. After trituration with a P200 pipet tip, 20 µl lysate was transferred to each of 3× wells of a clear flat-bottom 96-well plate as technical triplicates for each transfection condition. Luminescence was measured using a BioTek Synergy HT plate reader with the following settings: 10 s integration time, 135 gain, 1 mm read height. 100 µl/well Luciferase Assay Reagent II was dispensed into all wells containing cell lysate using P200 multichannel pipet and plate was immediately placed in plate reader. After firefly luciferase luminescence was measured, 100 µl/well Stop & Glo reagent was dispensed using P200 multichannel pipet and plate was immediately placed in plate reader. The fold-change relative to ‘no protein control’ was calculated by comparing ratios of firefly/*Renilla* luminescence from conditions ‘SIX1’ or ‘SIX1-Q177R’ to that of ‘no protein control’.

### Recombinant protein expression and purification

The same SIX1 and SIX1-Q177R gBlocks used for pCIG cloning were PCR amplified to add flanking BamHI and XhoI restriction sites. Digested gBlocks were ligated to digested pGEX-6p1-N-HA (gift from Andrew Jackson and Martin Reijns, Addgene plasmid #119756; RRID:Addgene_119756). BL21 (DE3) Competent *E. coli* (New England Biolabs) were transformed following manufacturer's protocol and streaked LB agar+1× ampicillin plates were incubated overnight at 37°C. One colony was picked from each transformation and cultured in 14 ml tubes each containing 7 ml LB broth+1× ampicillin, in orbital shaker at 37°C overnight. Glycerol stocks were made from each overnight liquid culture by mixing 50% glycerol solution and liquid bacteria culture 1:1, then stored at −80°C. A pipet tip was used to transfer a small amount of each glycerol stock to flasks containing 50 ml LB broth+1× ampicillin and cultures were incubated on orbital shaker overnight at 37°C. Each 50 ml culture was transferred to a 2-l flask containing 950 ml LB broth+1× ampicillin, and cultured at 37°C on orbital shaker until OD_600_=0.55–0.56. To each culture, 5 ml of 100 mM IPTG (Sigma-Aldrich) solution was added and flasks were incubated on an orbital shaker at 25°C for 19 h. For each culture, the entire culture volume was distributed into four 250-ml centrifuge bottles and centrifuged at 4640 ***g*** for 20 min at 4°C. All pellets from each culture were resuspended, pooled in 35 ml supernatant and the final suspension was transferred to 50 ml tubes. Tubes were centrifuged at 4000 ***g*** for 18 min at 4°C. Supernatants were discarded and pellets stored at −80°C.

Frozen bacteria pellets were thawed on ice and loosened in 25 ml lysis buffer [20 mM Tris-HCl, 150 mM NaCl, 1% Triton X-100, 10 mM DTT, 1× Protease Inhibitor Cocktail (Roche)]. Samples were sonicated on ice in a 4°C cold room using a Branson Sonifier 250, ten cycles of 10 s ON/OFF at 50% amplitude/duty cycle. Tubes were incubated on ice for 15 min then centrifuged at 13,000 ***g*** for 15 min at 4°C. H_2_O was decanted from a glutathione-agarose bead mixture (0.84 g glutathione-agarose beads in 168 ml H_2_O, incubated at 4°C overnight). 120 ml lysis buffer was added to the beads, mixed and incubated at 4°C for 20 min. Lysis buffer was decanted from the beads and 8 ml of bead slurry transferred to each of 2×50 ml tubes. Bacterial supernatant was added to tubes containing beads, and tubes were incubated on a tube rotator at 4°C for 1.5 h. EconoColumns (BioRad) were wetted and washed with 1× column volume PBS+1% Triton X-100, and then emptied. The supernatant–bead mixture was added to columns and column spigots opened full to allow gravity flow. Just before the flow stopped, beads were washed four times with one full column volume of cleavage buffer [50 mM Tris-HCl, 1 mM EDTA, 1 mM DTT, 1% Triton X-100 (Fisher BioReagents)]. PreScission Protease (200 µl; Cytiva) was mixed with 9.8 ml cleavage buffer; 5 ml of this mix was then added to each column, inverted to mix, incubated at 4°C for 2 h, inverted to mix and incubated at 4°C for an additional 2 h. Flow-through was collected in 15 ml conical tubes. 12–14,000 Da molecular weight cut-off (MWCO) dialysis tubing (Spectrum Laboratories) was incubated at RT in 1.5 l H_2_O+EDTA (5 mM) for 2–3 h, then rinsed thoroughly with H_2_O.

The entire volume of one flow-through was transferred to dialysis tubing, ends clipped shut and incubated overnight at 4°C submerged in 1 l dialysis buffer (50 mM Tris-HCl, 1 mM EDTA, 0.8 mM DTT) with gentle stirring. Used dialysis buffer was discarded, replaced with 1 l fresh dialysis buffer and incubation was continued at 4°C for 2.5 h. The contents of each dialysis tubing was transferred to 15-ml conical tubes. Membranes of 4× Amicon Ultra-4 30,000 Da MWCO Centrifugal Filter Units (Millipore) were pre-washed with H_2_O and then removed. For each protein solution, 2.5 ml was transferred to each of 2× filter units. Tubes were centrifuged at 3000 ***g*** for 25 min at 4°C. For each protein concentrate, all volumes were pooled from filter units. Protein concentration was measured using Pierce BCA Assay Kit (Thermo Scientific) and following the manufacturer's protocol with the following changes: in 96-well plate BSA controls were loaded in individual wells, and 10 µl of a 1:10 protein sample dilution was mixed with 190 µl working reagent in duplicate, added to individual wells, and the plate was incubated at 37°C for 30 min. Absorbance was measured at 562 nm on BioTek Synergy HT plate reader. BSA controls were used to generate a standard curve and the protein concentration of each purified protein sample was calculated. Dialysis buffer was added to each protein solution to bring concentrations to 2 mg/ml; aliquots were stored at −80°C. Protein purification was validated by SDS-PAGE, using a dilution series of each protein solution, followed by western blot using anti-SIX1 antibody (Cell Signaling Technology #12891) (Fig. S5).

### Microarray gene expression analysis

Raw CEL files were obtained from the GEO data repository accession number GSE53224, tumor histology was classified as in Wegert et al. (2015). Files were read into R using the oligo package (Carvalho and Irizarry, 2010). The package arrayQualityMetrics was used to identify and remove outlier samples (Kauffmann et al., 2009). RMA normalization was performed using the oligo package and annotation was performed using the hgu133plus2.db package (Carlson, 2016). Probes with suffixes other than ‘_a’ and ‘_a_at’, as well as duplicate probes, were manually removed, leaving only the highest expressing probe for each gene. The limma package was then used for differential expression analysis using the lmFit and eBayes functions (Smyth, 2005). Volcano plots of microarray data were made using the ‘Volcano Plot’ tool on the Galaxy web platform.

### Immunofluorescence and microscopy

A de-identified Wilms tumor tissue sample was obtained from the City of Hope Medical Center with exemption by the institutional review boards (Duarte, CA). The tissue sample was fixed in 4% paraformaldehyde for 10–30 min at RT in 1.5 ml centrifuge tube, followed two washes with PBS (5 min per wash). PBS was removed, ∼1 ml 30% sucrose solution in PBS was added and tube was placed on rocker overnight at 4°C. Once tissue sample no longer floated in 30% sucrose solution, it was washed 3× with 1 ml Optimal Cutting Temperature compound (OCT) to remove remaining sucrose (Fisher Health Care #4585). The tissue sample was then transferred to Biopsy Cryomold (Tissue-Tek) containing OCT, snap-frozen in 100% ethanol/dry ice mixture and stored at −80°C. The frozen block containing the tissue sample was sectioned at 12µm using a Leica CM1850 Cryostat and microscope slides containing tissue sections were stored at −20°C. Tissue section slides were thawed at RT and washed once in PBS to remove residual OCT. Sections were blocked in blocking solution (PBS+5% normal donkey serum+0.25% Triton X-100) for 30 min at RT. Primary antibody solutions were made in blocking solution and sections were incubated in primary antibody solution overnight at 4°C. Sections were washed 3× with wash buffer (PBS+0.25% Triton X-100). Secondary antibody solutions were made in blocking solution and sections were incubated in secondary antibody solution for 1 h at RT. Slides were washed 1× with wash buffer then 0.5 µg/ml DAPI solution was added to slides for 1–2 min. Slides were washed 3× with wash buffer and coverslips were mounted using Prolong Gold Antifade Mounting Medium. Stained slides were imaged using a Zeiss 880 Confocal Microscope with Airyscan (Plan-Neofluar 40×/1.3 oil WD0.21 objective or Plan-Apo 63×/1.4 oil objective). Antibodies used for immunofluorescence are listed in Table S4. Images were prepared for publication using Imaris Viewer software (Oxford Instruments).

### Electrophoretic mobility shift assays

Single-stranded DNA oligonucleotide probes were synthesized by IDT and then biotin end-labeled using the Pierce 3′ Biotin end-labeling DNA kit (Thermo Scientific) following the manufacturer's protocol with the following changes: 25 pmol oligonucleotide per reaction were labeled, reactions stopped with 1 µl 0.5M EDTA after 30 min. Complementary oligonucleotide labeling reactions were mixed prior to centrifugation at 13,000 ***g*** for 2 min. For unlabeled oligonucleotides, 50 µl H_2_O was mixed with 25 µl of each complementary 100 µM oligonucleotide. Annealing buffer was added to 1× and annealed following the same procedure as described for the luciferase assay. Annealed oligonucleotides were stored at −20°C.

Purified SIX1 and SIX1-Q177R recombinant proteins were diluted in H_2_O to the following molarities (nM): 1, 2, 4, 10, 20, 40, 100, 150, 200 and 300. EMSAs were performed using either Gelshift Chemiluminescent EMSA Kit (Active Motif) or LightShift Chemiluminescent EMSA Kit (Thermo Scientific) following manufacturer's protocol for setting up binding reactions, with the following changes: glycerol and poly d(I-C) were not included in reactions, 3 µl/reaction of 1:10 diluted biotin end-labeled probe was used, 12 µl/reaction H_2_O was used (13 µl for no protein control reaction), 1 µl of the appropriate protein dilution per reaction was added and reactions incubated at RT for 25 min. A 6% DNA Retardation Gel (Invitrogen) was pre-ran at 100V for 30 min in 0.5× TBE buffer (45 mM Tris base, 45 mM boric acid, 1 mM EDTA) in a Mini Gel Tank. Then, 5 µl of 5× loading dye was added to each reaction, 20 µl/reaction was loaded, and electrophoresis was performed at 100V for 1 h. Transfer was according to a wet-transfer protocol similar to that previously described for western blotting but using 0.5× TBE buffer as transfer buffer and transferring to Immobilin Ny+ nylon membrane (Millipore). Membranes were briefly put on a paper towel to dry. Membranes were placed face-down in a BioDoc-IT gel imager (UVP), UV lamp turned on, and incubated for 15 min to crosslink. Crosslinked membranes were either stored at −20°C or stained immediately following kit manufacturer's protocol. Stained membranes were imaged using iBright FL1500 Imaging System. ImageJ (Schneider et al., 2012) was used to obtain intensities of unbound and bound probe bands. The percentage of bound probe was calculated as follows: [bound probe intensity ÷ (bound probe intensity+unbound probe intensity)]×100.

### Mouse embryo collection for immunofluorescence

All animal studies were approved by the Office of Animal Care and Use at the University of North Carolina at Chapel Hill and the UNC Institutional Animal Care and Use Committee (IACUC). Procedures were performed under IACUC-approved protocol 22-136.0. Swiss Webster (Taconic stock SW, MPF) mice were utilized for timed matings. Plugs were ascertained by visual and probed inspection. The presence of a plug was considered to be day 0.5 of gestation. Embryos were collected at E10.5, fixed in 4% paraformaldehyde for ∼10 min then washed three times with PBS. Fixed embryos were embedded, cryosectioned, and sections were immunostained and imaged as described above under ‘Immunofluorescence and microscopy’.

## Supplementary Material

10.1242/dmm.050208_sup1 Supplementary information

Table S1. Microarray and RNA-seq differential gene expression analyses.

Table S2. Protein binding microarray data.

Table S3. SIX1/SIX1-Q177R Wilms tumor putative target genes and ChIP-seq peak coordinates.
