# Supplementary material for: Altered binding affinity of SIX1-Q177R correlates with enhanced WNT5A and WNT pathway effector expression in Wilms tumor
Source: Dis Model Mech. 2023 Nov 17;16(11):dmm050208. doi: 10.1242/dmm.050208 (PMC10668032; doi:10.1242/dmm.050208)
Supplement: Supplementary information [file dmm-16-050208-s1.pdf]

A)

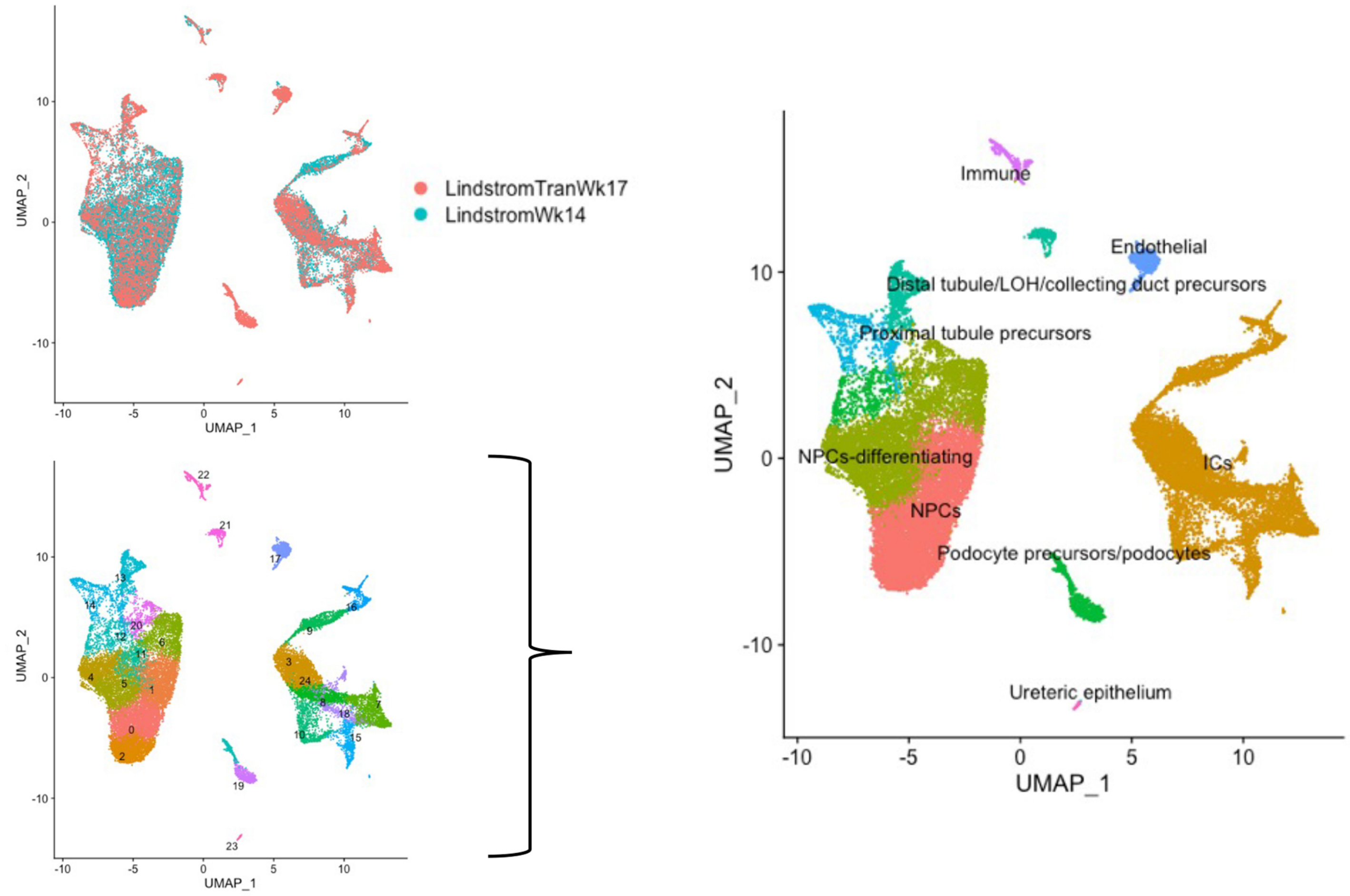

B)

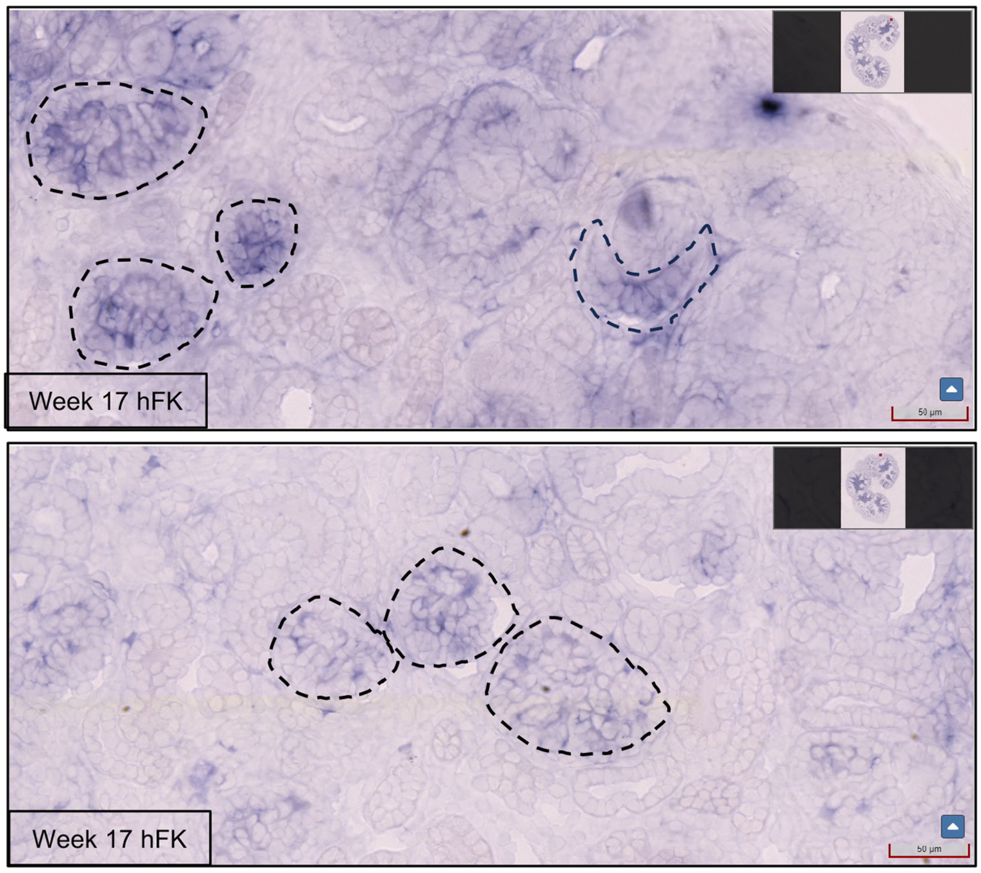

In situ hybridization images: Andrew McMahon (Re)Building a Kidney Consortium https://www.rebuildingakidney.org/id/16-QME2 (2019).

C)

Expression of upregulated SIX1/2-Q177R genes (compared to other blastemal tumors) in normal hFK

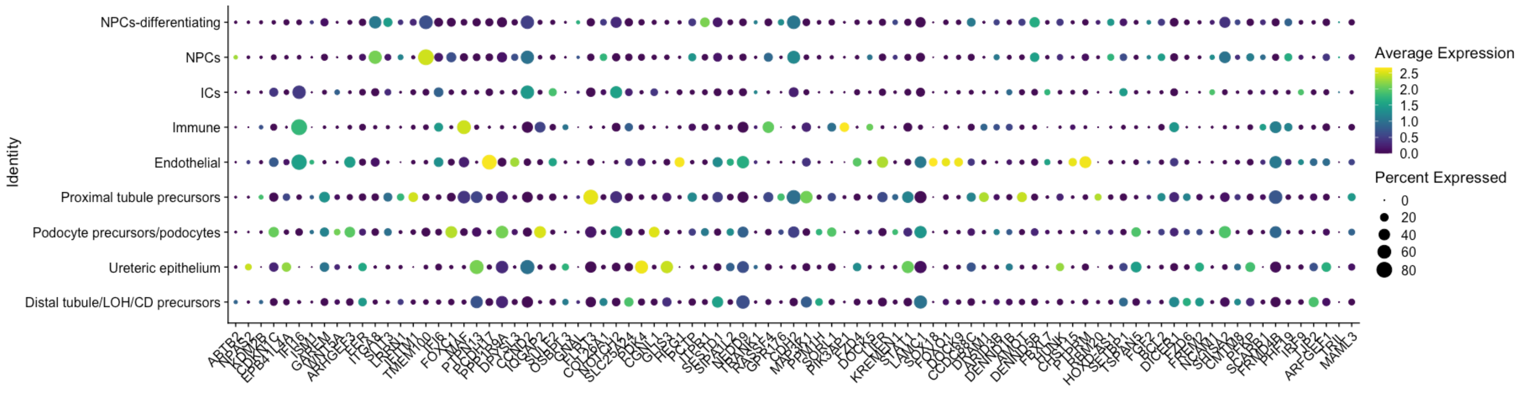

Fig. S1. A) Left: Top - UMAP plot showing locations of cells belonging to each of the indicated hFK single cell RNA-seq datasets (LindstromTranWk17 = GSE112570 and GSE124472 (only sample GSM3534656), LindstromWk14 = GSE139280). Bottom - 25 clusters generated from UMAP dimensional reduction (see Methods). Right: UMAP plot of same cells as left, but clusters were grouped and annotated based on known marker expression from literature (see Methods). **B)** Images of *WNT5A in situ* hybridization in week 17 hFK sections obtained from the GenitoUrinary Development Molecular Anatomy Project; https://www.rebuildingakidney.org/id/16-QME2. *WNT5A* signal is localized to podocytes in glomeruli (dashed circles) and podocyte precursors within a developing S-shaped body (dashed crescent). **C)** Dot plot generated from integrated hFK single-cell RNA-seq datasets (see Methods) displaying scaled average expression of all cells within the indicated cell clusters and the percent of cells in each cluster expressing the indicated genes.

Disease Models & Mechanisms • Supplementary information

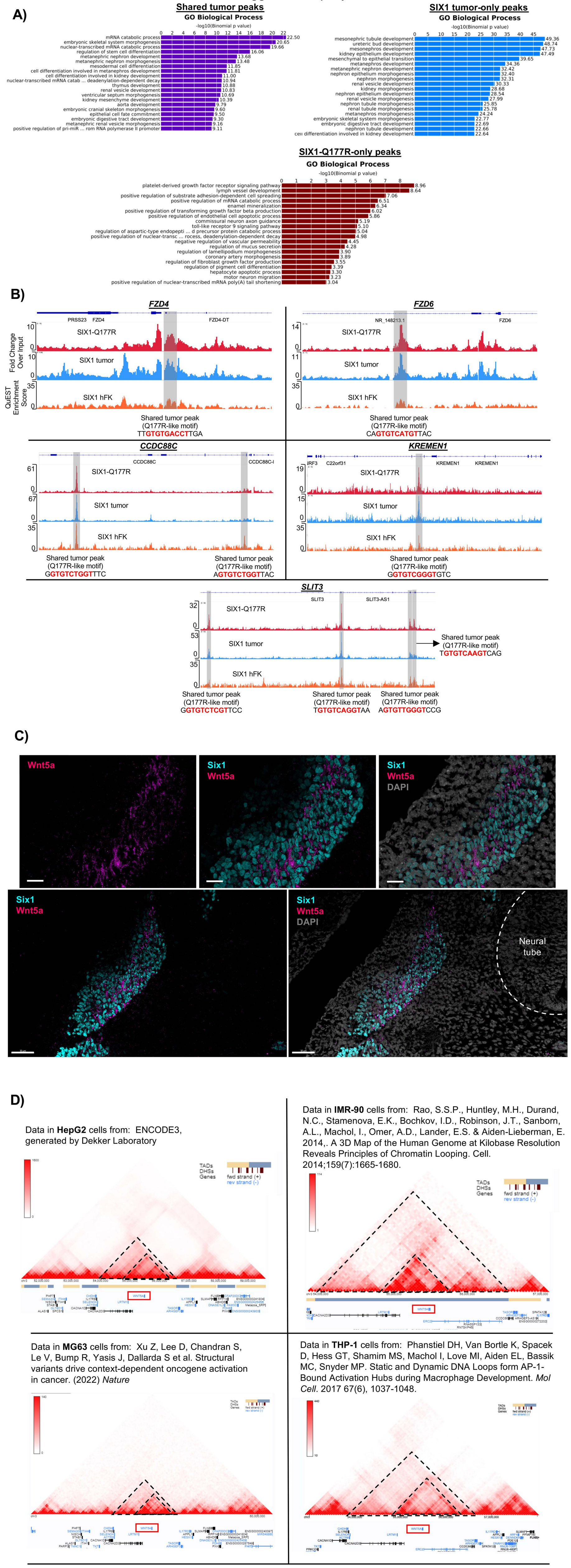
A)
Shared tumor peaks
GO Biological Process
-log10(Binomial p value)
SIX1 tumor-only peaks
GO Biological Process
-log10(Binomial p value)
SIX1-Q177R-only peaks
GO Biological Process
-log10(Binomial p value)
B)
FZD4
FZD6
Fold Change Over Input
QuEST Enrichment Score
SIX1-Q177R
SIX1 tumor
SIX1 hFK
Shared tumor peak
(Q177R-like motif)
TTGTGTGACCTTGA
CAGTGTCATGTTAC
CCDC88C
KREMEN1
GGTGTCTGGTTTC
AGTGTCTGGTTAC
GGTGTCGGGTGTC
SLIT3
TGTGTCAAGTCAG
GGTGTCTCGTTCC
TGTGTCAGGTAA
AGTGTTGGGTCCG
C)
Wnt5a
Six1
DAPI
Neural tube
D)
Data in HepG2 cells from: ENCODE3, generated by Dekker Laboratory
Data in IMR-90 cells from: Rao, S.S.P., Huntley, M.H., Durand, N.C., Stamenova, E.K., Bochkov, I.D., Robinson, J.T., Sanborn, A.L., Machol, I., Omer, A.D., Lander, E.S. & Aiden-Lieberman, E. 2014,. A 3D Map of the Human Genome at Kilobase Resolution Reveals Principles of Chromatin Looping. Cell. 2014;159(7):1665-1680.
Data in MG63 cells from: Xu Z, Lee D, Chandran S, Le V, Bump R, Yasis J, Dallarda S et al. Structural variants drive context-dependent oncogene activation in cancer. (2022) Nature
Data in THP-1 cells from: Phanstiel DH, Van Bortle K, Spacek D, Hess GT, Shamim MS, Machol I, Love MI, Aiden EL, Bassik MC, Snyder MP. Static and Dynamic DNA Loops form AP-1-Bound Activation Hubs during Macrophage Development. Mol Cell. 2017 67(6), 1037-1048.
TADs
DHSs
Genes
fwd strand (+)
rev strand (-)
WNT5A

Fig. S2. A) Bar charts displaying top 20 most significantly enriched biological processes for putative target genes associated with shared tumor, SIX1 tumor-only, and SIX1-Q177R tumor-only ChIP-seq peaks generated using GREAT (see methods). **B)** IGV genome browser snapshots displaying SIX1-Q177R tumor (red), SIX1 tumor (blue), and hFK SIX1 (orange) ChIP-seq tracks around the indicated gene loci. Genes shown were identified as upregulated in SIX1/2-Q177R tumors compared to other blastemal tumors in DGE analysis and are associated with WNT signaling. Shaded regions are peaks shared by both SIX1 and SIX1- Q177R in Wilms tumor containing DNA motifs that closely match the primary SIX1-Q177R binding motif. **C)** Section immunofluorescence images from E10.5 mouse embryos showing colocalization of Six1 and Wnt5a within the presumptive myotome. Top row scale bars = 20 µm. Bottom row scale bars = 50 µm. All images acquired on Zeiss 880 Confocal Microscope with Airyscan (Plan-Apo 63x/1.4 oil objective). **D)** Hi-C chromatin interaction heatmaps surrounding the *WNT5A* locus in the indicated cell lines. Source dataset for each heatmap is indicated above each plot. Heatmaps were downloaded from the 3D Genome Browser web tool, http://3dgenome.fsm.northwestern.edu/view.php (Wang et al., 2018). Blue and tan colored bars underneath heatmaps in HepG2 and IMR-90 panels indicate positions of TADs as labeled by the 3D Genome Browser web tool. Dashed triangles indicate regions of TADs corresponding to those identified in IMR-90 and HepG2 cells, as well as putative sub-TADs containing *WNT5A*.

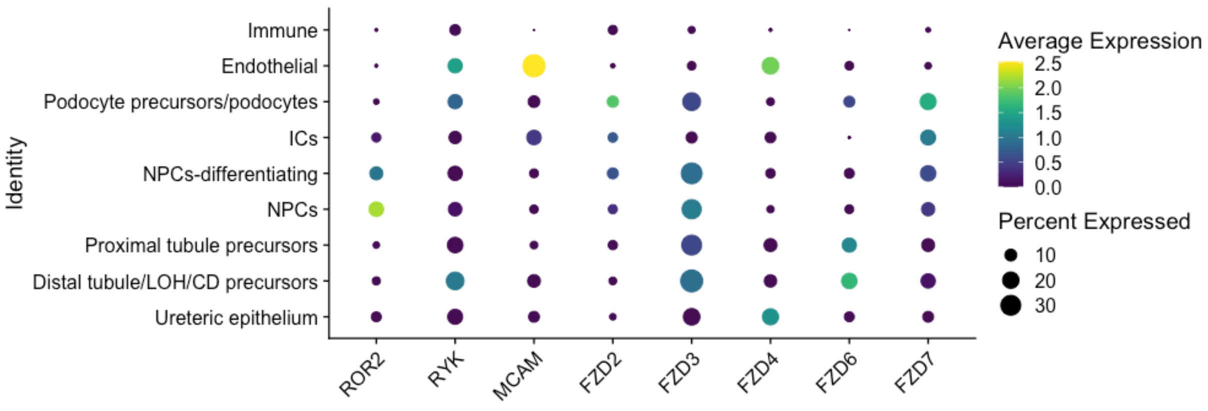

Fig. S3. Dot plot of genes encoding putative WNT5A receptors within the integrated hFK single-cell RNA-seq data displaying the scaled average expression value for each gene on the x-axis and the percent of cells expressing that gene within the cell clusters shown on the y-axis.

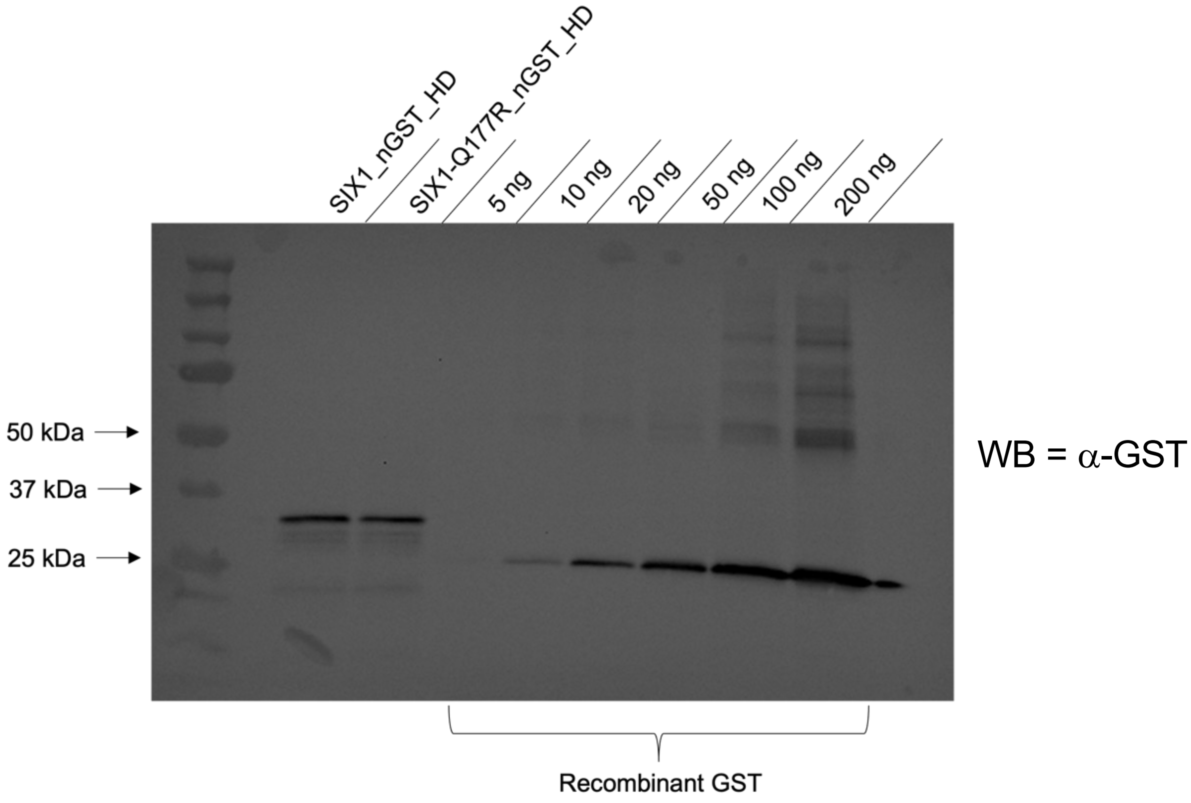

Fig. S4. Western Blot using α-GST antibody that was used to quantify concentration of ***in vitro*** transcribed/translated SIX1_nGST_HD and SIX1-Q177R_nGST_HD protein fragments alongside dilution series of recombinant GST protein for use in protein binding microarray assays.

Disease Models & Mechanisms • Supplementary information

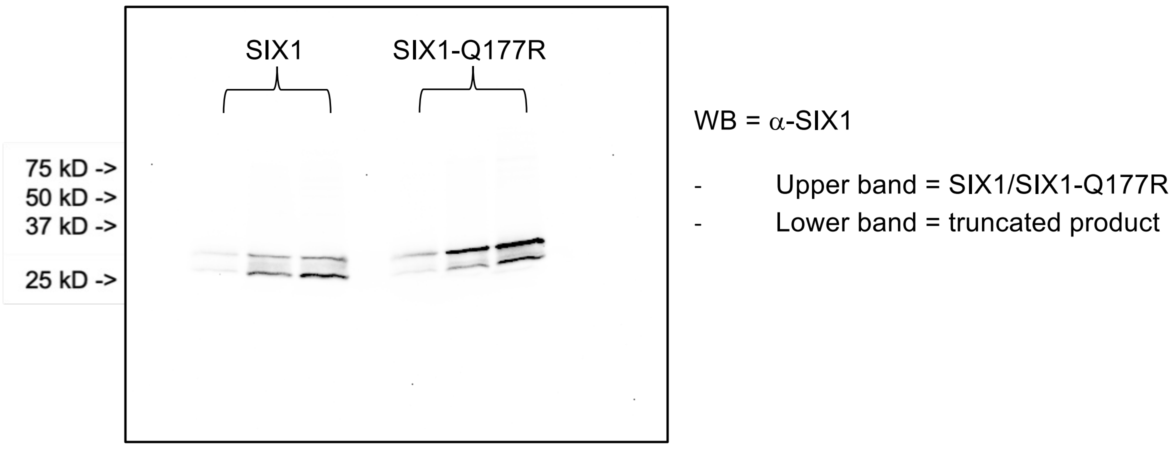

Fig. S5. Western blot using α-SIX1 antibody following SDS-PAGE of increasing concentrations of purified recombinant SIX1 and SIX1-Q177R.

Table S1. Microarray and RNA-seq differential gene expression analyses.

Available for download at
https://journals.biologists.com/dmm/article-lookup/doi/10.1242/dmm.050208#supplementary-data

Table S2. Protein binding microarray data.

Available for download at
https://journals.biologists.com/dmm/article-lookup/doi/10.1242/dmm.050208#supplementary-data

Table S3. SIX1/SIX1-Q177R Wilms tumor putative target genes and ChIP-seq peak coordinates.

Available for download at
https://journals.biologists.com/dmm/article-lookup/doi/10.1242/dmm.050208#supplementary-data

Table S4. Antibodies used for immunofluorescence

Antibody	Dilution
**WNT5A**<br>*R&D Systems #MAB645-SP*<br>References: Kaiser et al., 2019; Vesel et al 2017	1:50
**SIX1**<br>*Cell Signaling Technology #12891S*<br>References: O'Brien et al., 2016; Lindstrom et al., 2018c	1:500
**MAFB**<br>*R&D Systems #MAB3810*<br>References: Tran et al., 2019; Lindstrom et al., 2018d	1:500
**NCAM**<br>*Developmental Studies Hybridoma Bank #5.1H11*<br>References: Zhang et al., 2007; Mesires and Doumit 2002	1:20
**PODXL**<br>*R&D Systems #AF1658*<br>References: Morizane and Bonventre 2017; Ettou et al., 2020	1:1000

Table S5. Oligo and primer sequences used in cloning, luciferase assays, EMSAs, and PBMs

**>*SIX1*_enhancer_Luciferase assay**
ATTAGCTAGCCCAGCCGCGGCCCCAGCCCTCCCCCCAGCCTGTGCTGGGCTCCGCTTTCCCTCCATCAACTCCAAGCCGAATTCAA<br>TCCGAGAAGGCTCCTTTGAGCTTTTGTGTTTGCTGGGGGAGATGTGGGCGCAGGAGGGATCGCGTTACAACTTTCATTTCCTGAAAT<br>GTTTGAGGGAACATCCAGGGTTTTTATCCCCACATCAGGCCGGGCGATGGGCTCGAGTTTCAGGCCTTGTCACTCAGCTGTCACCAA<br>ACAAACGAAGCTCTCAGAGCCCAGGAGAGGGAGAGCTACCTGCTATTCATGACCCCTGGAGCAGGTGATCGCTCATGGGAAAAACA<br>GGTAGAATTAATCATAGGACTGTTCTCTGTTTCCTCTCCTTTTTGGCAGACCTGCCCACAGTGCGAAACCTATCAGCAAACAAATTAAC<br>TCTTTCTTGTCACCCAGGGGAATTAAAAATACGGTTTTAGAAAATAACTAAAACACACACGTTTCTCACCAAATGACAAAATGGGAGTT<br>GGAAGGAACATCTTACATCCCGCCCTACCTTGCAATTTCTTAGTTTGTGGGATTCTCATCTTGGTAGGATTTAGAACTTGGGGAAACT<br>GGTAGAGAATGAGGCATTCTCATGTTACCTGGTCCTACTACTGGATGGAGCCCCAGGTGTGGTCCCTGAACTCAGGTAGATTTCAAC<br>ATCTCCTTCTTGGCTGGGTGTCAATGCAGTTGGGCTTGATCCTACTAAAACAGAAAATCTCTACTCCAACAGGAATTTCTAAGAAATTT<br>CTGTAAATTAGTGTTTTGCTTCCCCCAGCCTCTTCCCTAAGTCAATCATGTTGAGCAAGCTTAATA

Disease Models & Mechanisms • Supplementary information

**>*WNT5A*_promoter_Luciferase assay**
TTATACCCAGCTAGCACGTAAGTCTAACCCTGCCGCACTGCATCGCCCATAGCCCCTGAAGGAGCCCCTTCCACAGAAAGAAAAGA<br>AAGGTGAGCCTCTTTAAGGCGGTGGAAGAGCCTGGCTTGGAACCTGTGCCGTAAAAGGGGCAGAGGGGACCTAGGCAGCCCTGGT<br>AAAGCTATGGGGCTCAGGGGCGTGCCAAGGTTTTTCTCCGTGAGCCGCCCCTTTGGCCTGGACGCTTCGGGGCTTCTCAAAGAGGA<br>AATGCTTATGTGGTCCCCAGCGCCTGCTAAGCAGGGCTCCACACCCAAGGCCCAGTTGTCCCCAAAACGCTGCAAAGCTGGGGGGC<br>GCATCCTGGAGAATGGAAATCTGGGGTTTCCCCAGCTAGGAGAGAGAAGGCTCCGGCTATCTCCCCACCCCCGCCCTAAGTGTCAA<br>ATTTCCTCCAGGGGAGGGAGTGGGCTGCAAAGTCTGCTTCTCGCGCAGCCCAGGCTGCAAAGTCAACTCTCCCCAAGGGCAGCCGA<br>TGCCGCGTGCACACACATCATACACATTCACACTCGTGCACATTTACACACTCACACGCGTGCATAGACACACACGCTGCGACATGT<br>TTCCGAGTCAGCGGCCCAGATTGGTGCTGGCCGCGTGCACTTTCCAAGCTTCGCGGCGAGCGGGGCGCGTGGGGCGGGGCTCAA<br>GCAGCAGAGAAATTGATAACAGATTCGGCGGATTACAGCGGATCTCTTTGTTAGAGCCGAAGCCACACAAACCGAACCCACTCCCAG<br>CCCGAAGCCCCCAGGGAGAGTCCACCAGTTCCCAGAGCCCAAAGCTTAATAGC
**>*WNT5A*_distal1_Luciferase assay**
TTATACCCAGCTAGCGTTTTATTAATTCCATGCTGGAGTTCCATAGCTGGGCCAGTCGGTTTGTAAGTTACCTCTGGAACAGTAGCTG<br>AACTACCCAGAGTGCATGATTGGAAAAGAGGAAATGGGGGAGGCTAGTCCTCCTCAACCTCTCACAGGAGAGAAATATGGTTTTCTC<br>CAGCTCCTACAGACAGGTTCCTGGGGCTATCTGGGGCTGGAGCCTCTAGGCAAGCTGATTTGAAACGCATCCAAGCGGATATCTTG<br>CTTCAAAGAGTCAGGCCCCCCCGGGGCAGAGCCTGAACCTGAGTACTGGAAGAGGACCAGGGGGCTGGAATTCAGGCACTGCCCT<br>CCCATCCCCCAGGCCCGGGAGGGTTTGTTTAACCCCTCAGGGGTGCTGTGTGCCTTCCTTGTCGAGGTGGTTTGCGGCTTCGTGAA<br>GCGATATTTATAGAGTGCTCTGTGATTCTTGGGTGAACATTCGTTTTATAAATGAATGATCAAATTTATTAGCAAAGAGTAACTAACCC<br>GTCTACATTTATTTTGTTGATAATACAAATTTACAAGGTTTTAATTGCCAGTTTTAAAACCCCCAAAACCCATAAATAAATACACGGGTA<br>ATAAAATATCTTCTCACTGTGCCCTTGACTTCTGTAGTTACATGAGGGCTTATGGGAATGTCTGAAGGAGCTTTCAGATGGAATCACAA<br>AACTTGTTTGAAAGGACTCAGGGCGGGAAACAGTCTAATTCAAACCTGGCTGGTGGTAATTTCATGTATGTTAACCAAAATGCTACTA<br>CTGATCATGAAGCTTAATAGC
**>*WNT5A*_distal2_Luciferase assay**
TTATACCCAGCTAGCGTGTACACACACGGTCGTGTTACAATTCTCATTTTGCAAAGTTTATGCAAAAACCAAACACCTGGGTTCAGAGT<br>TTCCTAAAGGAGTCATCTGAAGTAGGTGCTTTACGCCAAAACGTCACAAAGATTTATGTGCTTTCATTTGTGCATTAAATTGAGGACAG<br>GTGGGAGAATGCTCAGGCCTGAGAAAAACTGATAGCTCATTTCTCCCTTCGAAGAGAGATGGCTGTTATGACTACTGCTGGTTTAGAT<br>AAAATAGATACAGACTTTGTTTAAAAAAAAAAAAGAGGGCTCATGTTTGAAAAACAGTATTTCAGCAGTCAAATAATTACCTCTGCGAT<br>CATGTTTCTCACAAATGGAAAACCCTGGACTGAGATCCCACAGTGAGTCCTGCTCCTAGTTCAAACAACAGGCAATACCATGCCCAAC<br>AGCCAAGAAAATGGCCGGACCTCCCTTCACACTTGCTGAGGAAGGGTCCCTGGAATTGAGGCAAATGGTGGCCTCAACACATTCCAT<br>TTTAATGGCTTGCAACAGAGTCAGTTACACATATGTAACCACTCACTTTTTAATTTGATTCTTGTTTCAAACACCTTTTCAGGACCCAAT<br>ACATCTAAAAAATGTCATCACTTGATAGGTCGCTACTCTATCCCTGGCCTTGAAACAAGTGATTCGATGTGCTGAGTATTCACCAGGAT<br>TTGAAATAAGACTGTGAATTTGCATTTCAGAAAGGGAATACTATTTGGAAAGCCCAGTCTGCCAGACTTTTCACAAAGGGAAAGGAGA<br>GCTCCACTGGGAAAGCCTGCTGGTCAGCCAAGCTTAATAGC
**>*WNT5A*_promoter_EMSA**
CCCGCCCTAAGTGTCAAATTTCCTCCAGGGGAG
GGGCGGGATTCACAGTTTAAAGGAGGTCCCCTC
**>*WNT5A*_promoter_EMSAmut**
CCCGCCCTAAGT**A**TCAAATTTCCTCCAGGGGAG
GGGCGGGATTCA**T**AGTTTAAAGGAGGTCCCCTC
**>SIX1_gBlock for protein purification and luciferase assay**
GTTGTTCTCGAGGCCGCCACCATGTCGATGCTGCCGTCGTTTGGCTTTACGCAGGAGCAAGTGGCGTGCGTGTGCGAGGTTCTGCA<br>GCAAGGCGGAAACCTGGAGCGCCTGGGCAGGTTCCTGTGGTCACTGCCCGCCTGCGACCACCTGCACAAGAACGAGAGCGTACTC<br>AAGGCCAAGGCGGTGGTCGCCTTCCACCGCGGCAACTTCCGTGAGCTCTACAAGATCCTGGAGAGCCACCAGTTCTCGCCTCACAA<br>CCACCCCAAACTGCAGCAACTGTGGCTGAAGGCGCATTACGTGGAGGCCGAGAAGCTGCGCGGCCGACCCCTGGGCGCCGTGGG<br>CAAATATCGGGTGCGCCGAAAATTTCCACTGCCGCGCACCATCTGGGACGGCGAGGAGACCAGCTACTGCTTCAAGGAGAAGTCGA<br>GGGGTGTCCTGCGGGAGTGGTACGCGCACAATCCCTACCCATCGCCGCGTGAGAAGCGGGAGCTGGCCGAGGCCACCGGCCTCA<br>CCACCACCCAGGTCAGCAACTGGTTTAAGAACCGGAGGCAAAGAGACCGGGCCGCGGAGGCCAAGGAAAGGGAGAACACCGAAAA<br>CAATAACTCCTCCTCCAACAAGCAGAACCAACTCTCTCCTCTGGAAGGGGGCAAGCCGCTCATGTCCAGCTCAGAAGAGGAATTCTC<br>ACCTCCCCAAAGTCCAGACCAGAACTCGGTCCTTCTGCTGCAGGGCAATATGGGCCACGCCAGGAGCTCAAACTATTCTCTCCCGG<br>GCTTAACAGCCTCGCAGCCCAGTCACGGCCTGCAGACCCACCAGCATCAGCTCCAAGACTCTCTGCTCGGCCCCCTCACCTCCAGT<br>CTGGTGGACTTGGGGTCCTAAGGATCCTTGTTG
**CONTINUED ON NEXT PAGE**

Disease Models & Mechanisms • Supplementary information

**>SIX1-Q177R_gBlock for protein purification and luciferase assay**
GTTGTTCTCGAGGCCGCCACCATGTCGATGCTGCCGTCGTTTGGCTTTACGCAGGAGCAAGTGGCGTGCGTGTGCGAGGTTCTGCA<br>GCAAGGCGGAAACCTGGAGCGCCTGGGCAGGTTCCTGTGGTCACTGCCCGCCTGCGACCACCTGCACAAGAACGAGAGCGTACTC<br>AAGGCCAAGGCGGTGGTCGCCTTCCACCGCGGCAACTTCCGTGAGCTCTACAAGATCCTGGAGAGCCACCAGTTCTCGCCTCACAA<br>CCACCCCAAACTGCAGCAACTGTGGCTGAAGGCGCATTACGTGGAGGCCGAGAAGCTGCGCGGCCGACCCCTGGGCGCCGTGGG<br>CAAATATCGGGTGCGCCGAAAATTTCCACTGCCGCGCACCATCTGGGACGGCGAGGAGACCAGCTACTGCTTCAAGGAGAAGTCGA<br>GGGGTGTCCTGCGGGAGTGGTACGCGCACAATCCCTACCCATCGCCGCGTGAGAAGCGGGAGCTGGCCGAGGCCACCGGCCTCA<br>CCACCACCCAGGTCAGCAACTGGTTTAAGAACCGGAGGAGAAGAGACCGGGCCGCGGAGGCCAAGGAAAGGGAGAACACCGAAAA<br>CAATAACTCCTCCTCCAACAAGCAGAACCAACTCTCTCCTCTGGAAGGGGGCAAGCCGCTCATGTCCAGCTCAGAAGAGGAATTCTC<br>ACCTCCCCAAAGTCCAGACCAGAACTCGGTCCTTCTGCTGCAGGGCAATATGGGCCACGCCAGGAGCTCAAACTATTCTCTCCCGG<br>GCTTAACAGCCTCGCAGCCCAGTCACGGCCTGCAGACCCACCAGCATCAGCTCCAAGACTCTCTGCTCGGCCCCCTCACCTCCAGT<br>CTGGTGGACTTGGGGTCCTAAGGATCCTTGTTG
**>EYA1-2xHA_Fwd primer for cloning**
GTTGTTGAATTCGCCGCCACCATGGAAATGCAGGATCTAACCA
**>EYA1-2xHA_Rev primer for cloning**
GTTGTTCTAGATTAAGCGTAATCTGGAACATCGTATGGGTAAGCGTAATCTGGAACATCGTATGGGTACAGGTACTCTAATTCCAAGG
**>SIX1_nGST_HD for PBM**
CATATGATGTCCCCTATACTAGGTTATTGGAAAATTAAGGGCCTTGTGCAACCCACTCGACTTCTTTTGGAATATCTTGAAGAAAAATA<br>TGAAGAGCATTTGTATGAGCGCGATGAAGGTGATAAATGGCGAAACAAAAAGTTTGAATTGGGTTTGGAGTTTCCCAATCTTCCTTAT<br>TATATTGATGGTGATGTTAAATTAACACAGTCTATGGCCATCATACGTTATATAGCTGACAAGCACAACATGTTGGGTGGTTGTCCAAA<br>AGAGCGTGCAGAGATTTCAATGCTTGAAGGAGCGGTTTTGGATATTAGATACGGTGTTTCGAGAATTGCATATAGTAAAGACTTTGAA<br>ACTCTCAAAGTTGATTTTCTTAGCAAGCTACCTGAAATGCTGAAAATGTTCGAAGATCGTTTATGTCATAAAACATATTTAAATGGTGAT<br>CATGTAACCCATCCTGACTTCATGTTGTATGACGCTCTTGATGTTGTTTTATACATGGACCCAATGTGCCTGGATGCGTTCCCAAAATT<br>AGTTTGTTTTAAAAAACGTATTGAAGCTATCCCACAAATTGATAAGTACTTGAAATCCAGCAAGTATATAGCATGGCCTTTGCAGGGCT<br>GGCAAGCCACGTTTGGTGGTGGCGACCATCCTCCAAAATATCGGGTGCGCCGAAAATTTCCACTGCCGCGCACCATCTGGGACGGC<br>GAGGAGACCAGCTACTGCTTCAAGGAGAAGTCGAGGGGTGTCCTGCGGGAGTGGTACGCGCACAATCCCTACCCATCGCCGCGTG<br>AGAAGCGGGAGCTGGCCGAGGCCACCGGCCTCACCACCACCCAGGTCAGCAACTGGTTTAAGAACCGGAGGCAAAGAGACCGGG<br>CCGCGGAGGCCAAGGAAAGGGAGAACACCGAAAACAATAACTCCTCCTCCAACTAACTCGAG
**>SIX1-Q177R_nGST_HD for PBM**
CATATGATGTCCCCTATACTAGGTTATTGGAAAATTAAGGGCCTTGTGCAACCCACTCGACTTCTTTTGGAATATCTTGAAGAAAAATA<br>TGAAGAGCATTTGTATGAGCGCGATGAAGGTGATAAATGGCGAAACAAAAAGTTTGAATTGGGTTTGGAGTTTCCCAATCTTCCTTAT<br>TATATTGATGGTGATGTTAAATTAACACAGTCTATGGCCATCATACGTTATATAGCTGACAAGCACAACATGTTGGGTGGTTGTCCAAA<br>AGAGCGTGCAGAGATTTCAATGCTTGAAGGAGCGGTTTTGGATATTAGATACGGTGTTTCGAGAATTGCATATAGTAAAGACTTTGAA<br>ACTCTCAAAGTTGATTTTCTTAGCAAGCTACCTGAAATGCTGAAAATGTTCGAAGATCGTTTATGTCATAAAACATATTTAAATGGTGAT<br>CATGTAACCCATCCTGACTTCATGTTGTATGACGCTCTTGATGTTGTTTTATACATGGACCCAATGTGCCTGGATGCGTTCCCAAAATT<br>AGTTTGTTTTAAAAAACGTATTGAAGCTATCCCACAAATTGATAAGTACTTGAAATCCAGCAAGTATATAGCATGGCCTTTGCAGGGCT<br>GGCAAGCCACGTTTGGTGGTGGCGACCATCCTCCAAAATATCGGGTGCGCCGAAAATTTCCACTGCCGCGCACCATCTGGGACGGC<br>GAGGAGACCAGCTACTGCTTCAAGGAGAAGTCGAGGGGTGTCCTGCGGGAGTGGTACGCGCACAATCCCTACCCATCGCCGCGTG<br>AGAAGCGGGAGCTGGCCGAGGCCACCGGCCTCACCACCACCCAGGTCAGCAACTGGTTTAAGAACCGGAGGAGAAGAGACCGGG<br>CCGCGGAGGCCAAGGAAAGGGAGAACACCGAAAACAATAACTCCTCCTCCAACTAACTCGAG
**>Minimal_Promoter1 for pBV-Luc**
AGTAAGCTTGGGGGTATATAATGGATCCGGTATCGAGATCTGCGATCTAAGTAAGTTGGCATTCCGGTACTGTTAAAGCCACCATGG<br>AAC
**>Minimal_Promoter2 for pBV-Luc**
GTTCCATGGTGGCTTTAACAGTACCGGAATGCCAACTTACTTAGATCGCAGATCTCGATACCGGATCCATTATATACCCCCAAGCTTA<br>CT

Disease Models & Mechanisms • Supplementary information

SUPPLEMENTAL REFERENCES

Ettou, S., Jung, Y. L., Miyoshi, T., Jain, D., Hiratsuka, K., Schumacher, V., Taglienti, M., Morizane, R., Park, P. J., & Kreidberg, J. A. (2020). Epigenetic transcriptional reprogramming by WT1 mediates a repair response during podocyte injury. *Science Advances*, ***6*****(30)**. https://doi.org/10.1126/sciadv.abb5460

Kaiser, K., Gyllborg, D., Prochazka, J., Salašová, A., Kompanikova, P., Molina, F. L., Laguna-Goya, R., Radaszkiewicz, T., Harnoš, J., Prochazkova, M., et al. (2019). WNT5A is transported via lipoprotein particles in the cerebrospinal fluid to regulate hindbrain morphogenesis. *Nature Communications*, ***10*****(1)**. https://doi.org/10.1038/s41467-019-09298-4

Mesires, N. T., & Doumit, M. E. (2002). Satellite cell proliferation and differentiation during postnatal growth of porcine skeletal muscle. *American Journal of Physiology-cell Physiology*, ***282*****(4)**, C899–C906. https://doi.org/10.1152/ajpcell.00341.2001

Morizane, R., & Bonventre, J. V. (2016). Generation of nephron progenitor cells and kidney organoids from human pluripotent stem cells. *Nature Protocols*, ***12*****(1)**, 195–207. https://doi.org/10.1038/nprot.2016.170

Vesel, M., Rapp, J., Feller, D., Kiss, E., Jaromi, L., Meggyes, M., Miskei, G., Duga, B., Smuk, G., László, T., Karner, I., & Pongracz, J. E. (2017). ABCB1 and ABCG2 drug transporters are differentially expressed in non-small cell lung cancers (NSCLC) and expression is modified by cisplatin treatment via altered Wnt signaling. *Respiratory Research*, ***18*****(1)**. https://doi.org/10.1186/s12931-017-0537-6

Zhang, F., Pomerantz, J. H., Sen, G. L., Palermo, A. T., & Blau, H. M. (2007). Active tissue-specific DNA demethylation conferred by somatic cell nuclei in stable heterokaryons. *Proceedings of the National Academy of Sciences*, ***104*****(11)**, 4395–4400. https://doi.org/10.1073/pnas.0700181104
